# Supplementary material for: Phertilizer: Growing a clonal tree from ultra-low coverage single-cell DNA sequencing of tumors
Source: PLoS Comput Biol. 2023 Oct 11;19(10):e1011544. doi: 10.1371/journal.pcbi.1011544 (PMC10593221; doi:10.1371/journal.pcbi.1011544)
Supplement: S1 Appendix — (PDF) [file pcbi.1011544.s001.pdf]

Supplementary Text — Phertilizer: Growing a clonal tree from ultra-low coverage single-cell DNA sequencing of tumors

Leah L. Weber [1,*] Chuanyi Zhang [2,*] Idoia Ochoa [2,3,†] Mohammed El-Kebir [1,4,†]

[1]Dept. of Computer Science, University of Illinois Urbana-Champaign, IL, USA
[2]Dept. of Electrical & Computer Engineering, University of Illinois Urbana-Champaign, IL, USA
[3]Dept. of Electrical and Electronics Engineering, University of Navarre, Donostia, Spain
[4]Cancer Center at Illinois, University of Illinois Urbana-Champaign, IL, USA

[*]Shared first authorship; [†]Corresponding author: {idoia,melkebir}@illinois.edu

Contents

A Supplementary methods **2**
A.1 Data processing . . . 2
A.2 Generative model . . . 2
A.2.1 Latent variant allele frequency model . . . 3
A.2.2 Variant read count model . . . 3
A.2.3 Binned read count embedding model . . . 4
A.2.4 Posterior probability . . . 5
A.3 Detectability of a clone . . . 6
A.4 PHERTILIZER . . . 8
A.4.1 Growing phase . . . 8
A.4.2 `Linear` . . . 10
A.4.3 `Branching` . . . 12
A.4.4 `Identity` . . . 14
A.4.5 Running time of an elementary tree operation . . . 14
A.4.6 Regularization . . . 14
A.4.7 Postprocessing . . . 15

B Supplementary results **16**
B.1 Simulation study . . . 16
B.1.1 BASELINE method . . . 16
B.1.2 Simulation setup . . . 16
B.1.3 Runtime parameters . . . 17
B.1.4 Performance metrics . . . 18
B.1.5 Supplemental simulation study figures . . . 19
B.2 Experimental data . . . 27
B.2.1 PHERTILIZER runtime parameters for experimental data . . . 27
B.2.2 Placement of driver genes on inferred trees . . . 27
B.2.3 Cell mutational burden (CMB) . . . 27
B.2.4 Supplemental figures for high-grade serous ovarian cancer cells sequenced with DLP+ 30

B.2.5 Processing the triple negative breast cancer tumors sequencing data from ACT . . . 31
B.2.6 Supplemental tables and figures for triple negative breast cancer tumors sequenced with ACT . . . 31

List of Figures

A Plate diagram . . . 2
B Visual depiction of the detectability of a clone . . . 7
C A graphical depiction of PHERTILIZER's recursive enumeration tree building process . . . 10
D A graphical depiction of a `Linear` elementary tree operation . . . 11
E A graphical depiction of a `Branching` elementary tree operation . . . 12
F Genotype similarity between inferred genotypes per cell cluster using the BASELINE method and varying the variant allele frequency (VAF) threshold and inferred clonal genotypes per cell cluster by Laks et al. [1] . . . 17
G Example for ancestral pair recall (APR), clustered pair recall (CPR), incomparable pair recall (IPR), and accuracy for cells . . . 18
H Simulation results for coverage $g = 0.01\times$ . . . 20
I Simulation results for coverage $g = 0.05\times$ . . . 21
J Simulation results for coverage $g = 0.1\times$ . . . 22
K Heterozygous diploid simulation results aggregrated over $k \in \{5, 9\}$ clones and $m \in \{5000, 10000, 15000\}$ SNVs . . . 23
L Dollo evolutionary model simulation results for $k = 9$ clones and $m = 15000$ SNVs . . . 24
M Running time (in seconds) on simulation data . . . 24
N Hyperparameter impact on genotype similarity . . . 25
O Hyperparameter impact on tree reconstruction accuracy . . . 26
P Analysis of cell mutational burden (CMB) on a simulated instance with varying error rates in $\{0\%, 15\%, 30\%\}$ for both cell and SNV placement . . . 29
Q UMAP for the high-grade serous ovarian cancer patient depicted with inferred cell clusterings 30
R Cell clustering comparison for triple negative breast tumor TN1 in embedding space between PHERTILIZER and Minussi et al. [2] . . . 32
S Cell mutational burden per clade in trees inferred by BASELINE+SCITE for tumors TN3 and TN5 . . . 33
T PHERTILIZER inferred clonal tree for breast cancer tumor TN2 . . . 33
U PHERTILIZER inferred clonal tree for breast cancer tumor TN4 . . . 34
V PHERTILIZER inferred clonal tree for breast cancer tumor TN8 . . . 34

List of Tables

A Summary of results on the Minussi et al. [2] breast cancer data . . . 31

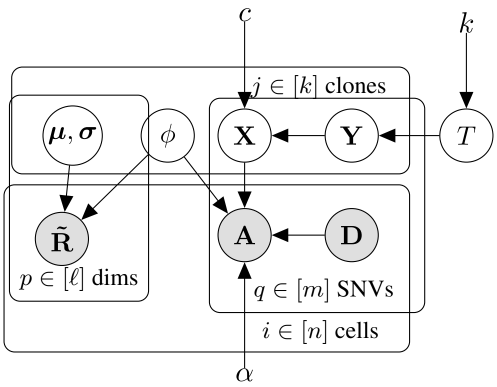

Fig A: **Plate diagram.** The observed data consists of variant and total read counts $\mathbf{A}, \mathbf{D}$ and binned read counts $\tilde{\mathbf{R}}$, generated from an unobserved cell clustering ϕ and clonal tree T of k clones with clonal genotypes $\mathbf{Y}$. Given hyperparameters $\theta = (c, \alpha)$ and observed data $(\mathbf{A}, \mathbf{D}, \tilde{\mathbf{R}})$, we seek $(T, \mathbf{Y}, \phi)$ with maximum posterior probability $P(T, \mathbf{Y}, \phi, \mid \mathbf{A}, \mathbf{D}, \tilde{\mathbf{R}}, \theta)$, marginalizing over latent variables $\mathbf{X}$, $\boldsymbol{\mu}$ and $\boldsymbol{\sigma}$.

A Supplementary methods

A.1 Data processing

PHERTILIZER either takes as input binned read counts $\mathbf{R} \in \mathbb{N}^{n \times b}$ of n cells with read counts grouped into b bins or alternatively a binned read count embedding $\tilde{\mathbf{R}} \in \mathbb{R}^{n \times \ell}$ of read counts of n cells projected into $\ell \ll b$ dimensional space. If provided the former, PHERTILIZER will project the binned read counts $\mathbf{R}$ into $\ell = 2$ dimensions as follows. First, we compute normalized binned read counts $R' = [r'_{ip}] \in \mathbb{R}^{n \times b}$ as

$$r'_{ip} = \frac{r_{ip}}{\sum_{p'=1}^{b} r_{ip'}}. \quad (1)$$

Next, we use UMAP [3] to project the normalized binned read counts $\mathbf{R}'$ to $\ell = 2$ dimensions with the following parameter settings:

```
n_neighbors=40, spread=1, n_components=2, min_dist=0.
```

This yields the binned read count embedding $\tilde{\mathbf{R}} \in \mathbb{R}^{n \times \ell}$ where $\ell = 2$.

A.2 Generative model

Our generative model (Fig. A) captures the evolution and the subsequent process of ultra-low coverage sequencing of n cells with m SNVs yielding our data $\mathbf{A}, \mathbf{D} \in \mathbb{N}^{n \times m}$ and $\tilde{\mathbf{R}} \in \mathbb{R}^{n \times \ell}$. For a number $k \in \mathbb{N}$ of clones, we generate a rooted tree T with k nodes with a uniform prior $P(T \mid k)$. We generate clonal genotypes $\mathbf{Y}$ for tree T under the infinite sites model with $P(\mathbf{Y} \mid T)$ uniformly distributed. We model the cell clustering $\phi : [k] \rightarrow 2^{[n]}$ with $P(\phi)$ distributed uniformly over all possible partitions of n cells into k clones/parts. The prior probability $P(\mathbf{D})$ of total read counts $\mathbf{D}$ is also uniform.

The generative model has three remaining components: the (i) latent variant allele frequency (VAF) model, (ii) the variant read count model and (iii) the binned read count embedding model. First, the latent VAFs $\mathbf{X} \in \mathbb{Q}^{k \times m}$ are dependent on the clonal genotypes $\mathbf{Y}$. Thus, whenever clonal genotype $y_{jq} = 0$ for SNV q of clone j, the latent VAF $x_{jq} = 0$. Otherwise, x_{jq} is the ratio of chromosomal copies that harbor SNV q to the total number of chromosomal copies of clone j. Appendix A.2.1 contains the precise definition of $P(\mathbf{X} \mid \mathbf{Y}, \theta)$. Second, given latent VAFs $\mathbf{X}$, total read counts $\mathbf{D}$, cell clustering ϕ and the probability α of

misreading a single nucleotide during sequencing, we model variant read counts with a binomial distribution that accounts for sequencing errors. Appendix A.2.2 provides the derivation of $P(\mathbf{A} \mid \mathbf{D}, \mathbf{X}, \phi, \theta)$. Third, we model the generation of the binned read counts embedding $\tilde{\mathbf{R}}$ using a Gaussian mixture model where $\tilde{r}_{ip} \mid \phi, \boldsymbol{\mu}, \boldsymbol{\sigma} \sim \mathcal{N}(\mu_p, \sigma_p^2)$. Latent variables $\boldsymbol{\mu} \in \mathbb{R}^{k \times \ell}$ and $\boldsymbol{\sigma} \in \mathbb{R}_{\geq 0}^{k \times \ell}$ are independent for each clone $j \in [k]$ and dimension $p \in [\ell]$. Appendix A.2.3 contains the definition of $P(\tilde{\mathbf{R}} \mid \phi, \boldsymbol{\mu}, \boldsymbol{\sigma})$.

This model requires two hyperparameters c and α, where $c \in \mathbb{N}$ is the upper bound on the total number of chromosomal copies at any locus in the genome and $\alpha \in [0, 1]$ is the probability of misreading a single nucleotide during sequencing (Fig. A). Given hyperparameters $\theta = (c, \alpha)$ and observed data $(\mathbf{A}, \mathbf{D}, \tilde{\mathbf{R}})$, we seek to identify latent variables $(T, \mathbf{Y}, \phi)$ with maximum posterior probability $P(T, \mathbf{Y}, \phi \mid \mathbf{A}, \mathbf{D}, \tilde{\mathbf{R}}, \theta)$. We show in Appendix A.2.4 that this can be approximated as $P(\mathbf{A} \mid \mathbf{D}, \mathbf{Y}, \phi, \theta) P(\tilde{\mathbf{R}} \mid \phi, \boldsymbol{\mu}, \boldsymbol{\sigma})$ taking $\boldsymbol{\mu}$ and $\boldsymbol{\sigma}$ as the maximum *a posteriori* estimates.

A.2.1 Latent variant allele frequency model

The latent variant allele frequency (VAF) x_{jq} denotes the fraction of chromosomal copies that harbor the SNV at locus q for clone j. Let $\bar{d}_{jq}$ be the number of chromosomal copies of the SNV locus q in clone j, and let $\bar{a}_{jq} \leq \bar{d}_{jq}$ be the number of chromosomal copies that harbor the SNV. Mathematically, we define the latent VAF as $x_{jq} = \bar{a}_{jq} / \bar{d}_{jq}$.

The latent VAF $x_{jq} \in \mathbb{Q}$ is conditioned on the latent clonal genotype $y_{jq} \in \{0, 1\}$. Given a pre-specified maximum copy number $c \in \mathbb{N} \setminus \{0\}$, we define the multiset S of latent VAFs associated with SNV state $y_{jq} = 1$ as

$$S = \{s/t \mid 1 \leq s \leq t \leq c\}. \tag{2}$$

We denote with $|\{x \in S\}|$ the number of occurrences of latent VAF x in the multiset S. Then, we have

$$P(x_{jq} \mid y_{jq}, \theta) = \begin{cases} 1, & \text{if } y_{jq} = 0, x_{jq} = 0, \\ \frac{|\{x_{jq} \in S\}|}{|S|}, & \text{if } y_{jq} = 1, x_{jq} > 0, \\ 0 & \text{otherwise.} \end{cases} \tag{3}$$

For example, if we have maximum copy number $c = 2$, the SNV is present (i.e., $y_{jq} = 1$), we have that $S = \{1/1, 1/2, 2/2\}$ and that $P(x_{jq} = 1/2 \mid y_{jq} = 1, \theta) = 1/3$.

Without assuming any prior information on the chromosomal copy numbers in each clone j, this model assumes that the latent VAF x_{jq} is directly proportional to the number of occurrences in multiset S. Using independence among clones $[k]$ and SNV loci $[m]$ when given clonal genotypes $\mathbf{Y}$, we have that

$$P(\mathbf{X} \mid \mathbf{Y}, \theta) = \prod_{j=1}^{k} \prod_{q=1}^{m} P(x_{jq} \mid y_{jq}, \theta). \tag{4}$$

A.2.2 Variant read count model

Given latent VAFs $\mathbf{X}$, total read counts $\mathbf{D}$ and cell clustering ϕ, we model alternate read counts $\mathbf{A}$ as a binomial distribution accounting for sequencing errors. While previous models for medium-to-high coverage scDNA-seq data specifically account for errors introduced during PCR amplification [4, 5], the lack of preamplification in the latest generation of ultra-low scDNA-seq technology restricts errors to base calling errors that occur during sequencing [1, 2]. Without sequencing error, the success probability for a variant read would be latent VAF x_{jq}. Let α be the probability of misreading a single nucleotide during sequencing. For a successful trial, meaning we observe a variant allele, we have two independent cases. In case I, we have no sequencing error ($1 - \alpha$ probability) and x_{jq} probability of reading a variant allele. In case I we

have $1 - x_{jq}$ probability of sequencing a reference allele and $\alpha/3$ probability of misreading the reference allele for the variant allele.

Next we derive $P(\mathbf{A} \mid \mathbf{D}, \mathbf{X}, \phi, \theta)$.

$$P(a_{iq} \mid d_{iq}, \mathbf{x}_{\cdot q}, \phi, \theta) = \text{Binom}\left(a_{iq};\, d_{iq},\, x_{jq}(1-\alpha) + \frac{(1-x_{jq})\alpha}{3}\right), \tag{5}$$

where j is the unique clone corresponding to cell i, i.e., $i \in \phi(j)$. Using an indicator variable, this is equivalent to

$$P(a_{iq} \mid d_{iq}, \mathbf{x}_{\cdot q}, \phi, \theta) = \prod_{j=1}^{k} P(a_{iq} \mid d_{iq}, x_{jq}, \phi, \theta)^{\mathbf{1}\{i \in \phi(j)\}} \tag{6}$$

$$= \prod_{j=1}^{k} \left[\text{Binom}\left(a_{iq};\, d_{iq},\, x_{jq}(1-\alpha) + \frac{(1-x_{jq})\alpha}{3}\right)\right]^{\mathbf{1}\{i \in \phi(j)\}}. \tag{7}$$

Per the graphical model, we have independence among cells $[n]$ and SNV loci $[m]$ when conditioning on $\mathbf{D}, \mathbf{X}, \phi$ and θ, yielding

$$P(\mathbf{A} \mid \mathbf{D}, \mathbf{X}, \phi, \theta) = \prod_{j=1}^{k} \prod_{i=1}^{n} \prod_{q=1}^{m} P(a_{iq} \mid d_{iq}, x_{jq}, \phi, \theta)^{\mathbf{1}\{i \in \phi(j)\}} \tag{8}$$

$$= \prod_{j=1}^{k} \prod_{i=1}^{n} \prod_{q=1}^{m} \left[\text{Binom}\left(a_{iq};\, d_{iq},\, x_{jq}(1-\alpha) + \frac{(1-x_{jq})\alpha}{3}\right)\right]^{\mathbf{1}\{i \in \phi(j)\}}. \tag{9}$$

A.2.3 Binned read count embedding model

The projection of binned reads counts $\mathbf{R}$ into a low dimension embedding $\tilde{\mathbf{R}}$ has two advantages. First, it allows us to identify sets of cells with similar copy number profiles, without the overhead of copy number calling. Second, distance functions behave counter-intuitively in high dimensions [6, 7] and therefore dimensionality reduction provides us with a useful proxy for the distance between the copy number profile of pairs of cells. We model the generation of the binned read counts embedding $\tilde{\mathbf{R}}$ using a Gaussian mixture model where $\tilde{r}_{ip} \mid \phi, \boldsymbol{\mu}, \boldsymbol{\sigma} \sim \mathcal{N}(\mu_p, \sigma_p^2)$. Latent variables $\boldsymbol{\mu} \in \mathbb{R}^{k \times \ell}$ and $\boldsymbol{\sigma} \in \mathbb{R}_{\geq 0}^{k \times \ell}$ are independent for each clone $j \in [k]$ and dimension $p \in [\ell]$.

Following convention, let $z_i \in [k]$ be a random variable depicting the assignment of cell i to one of the k clones. Given a cell clustering ϕ, we have $P(z_i = j \mid \phi) = \mathbf{1}\{i \in \phi(j)\}$ meaning ϕ specifies a hard clustering of cells into k clones. Given cell clustering ϕ and latent variables $\boldsymbol{\mu}_j = [\mu_{jp}]$ and $\boldsymbol{\sigma}_{\boldsymbol{j}} = [\sigma_{jp}]$ for each clone $j \in [k]$, the probability of binned read count embedding for cell i and dimension p is thus

$$P(\tilde{r}_{ip}, \mid \phi, \boldsymbol{\mu}, \boldsymbol{\sigma}) = \sum_{j=1}^{k} P(z_i = j \mid \phi) \cdot \mathcal{N}(\mu_{jp}, \sigma_{jp}^2) \tag{10}$$

$$= \prod_{j=1}^{k} \mathcal{N}(\mu_{jp}, \sigma_{jp}^2)^{\mathbf{1}\{i \in \phi(j)\}}. \tag{11}$$

Thus, using independence among cells and dimensions, we have the following probability

$$P(\tilde{\mathbf{R}} \mid \phi, \boldsymbol{\mu}, \boldsymbol{\sigma}) = \prod_{j=1}^{k} \prod_{i=1}^{n} \prod_{p=1}^{\ell} \mathcal{N}(\mu_{jp}, \sigma_{jp}^2)^{\mathbf{1}\{i \in \phi(j)\}}. \tag{12}$$

A.2.4 Posterior probability

As discussed in Main Text, given hyperparameters $\theta = (c, \alpha)$ we seek to solve

$$\arg\max_{T,\mathbf{Y},\phi} P(T, \mathbf{Y}, \phi \mid \mathbf{A}, \mathbf{D}, \tilde{\mathbf{R}}, \theta). \tag{13}$$

This is equivalent to marginalizing over latent variables $\boldsymbol{\mu}$ and $\boldsymbol{\sigma}$,

$$\arg\max_{T,\mathbf{Y},\phi} \int_{\boldsymbol{\mu}} \int_{\boldsymbol{\sigma}} P(T, \mathbf{Y}, \phi, \boldsymbol{\mu}, \boldsymbol{\sigma} \mid \mathbf{A}, \mathbf{D}, \tilde{\mathbf{R}}, \theta)\, d\boldsymbol{\mu}\ d\boldsymbol{\sigma}. \tag{14}$$

Computing the integrals over latent variables $\boldsymbol{\mu}$ and $\boldsymbol{\sigma}$ is challenging in practice, so we instead approximate the above expression by searching for the maximum *a posteriori* (MAP) estimates for latent variables $(\boldsymbol{\mu}, \boldsymbol{\sigma})$, giving the expression

$$\arg\max_{T,\mathbf{Y},\phi,\boldsymbol{\mu},\boldsymbol{\sigma}} P(T, \mathbf{Y}, \phi, \boldsymbol{\mu}, \boldsymbol{\sigma} \mid \mathbf{A}, \mathbf{D}, \tilde{\mathbf{R}}, \theta). \tag{15}$$

We begin by applying Bayes' rule, obtaining

$$P(T, \mathbf{Y}, \phi, \boldsymbol{\mu}, \boldsymbol{\sigma}, \mid \mathbf{A}, \mathbf{D}, \tilde{\mathbf{R}}, \theta) = \frac{P(T, \mathbf{Y}, \phi, \boldsymbol{\mu}, \boldsymbol{\sigma} \mid \theta) P(\mathbf{A}, \mathbf{D}, \tilde{\mathbf{R}} \mid T, \mathbf{Y}, \phi, \boldsymbol{\mu}, \boldsymbol{\sigma}, \theta)}{P(\mathbf{A}, \mathbf{D}, \tilde{\mathbf{R}} \mid \theta)}. \tag{16}$$

We decompose $P(T, \mathbf{Y}, \phi, \boldsymbol{\mu}, \boldsymbol{\sigma} \mid \theta)$ per the graphical model and obtain

$$P(T, \mathbf{Y}, \phi, \boldsymbol{\mu}, \boldsymbol{\sigma}, \mid \mathbf{A}, \mathbf{D}, \tilde{\mathbf{R}}, \theta) = \frac{P(T \mid \theta) P(\mathbf{Y} \mid T, \theta) P(\phi \mid \theta) P(\boldsymbol{\mu}, \boldsymbol{\sigma} \mid \theta) P(\mathbf{A}, \mathbf{D}, \tilde{\mathbf{R}} \mid T, \mathbf{Y}, \phi, \boldsymbol{\mu}, \boldsymbol{\sigma}, \theta)}{P(\mathbf{A}, \mathbf{D}, \tilde{\mathbf{R}} \mid \theta)}. \tag{17}$$

Using uniformity of $P(T \mid \theta)$, $P(\mathbf{Y} \mid T, \theta)$, $P(\phi \mid \theta)$, $P(\boldsymbol{\mu}, \boldsymbol{\sigma} \mid \theta)$ and dropping $P(\mathbf{A}, \mathbf{D}, \tilde{\mathbf{R}} \mid \theta)$ from the denominator, we obtain

$$P(T, \mathbf{Y}, \phi, \boldsymbol{\mu}, \boldsymbol{\sigma} \mid \mathbf{A}, \mathbf{D}, \tilde{\mathbf{R}}, \theta) \propto P(\mathbf{A}, \mathbf{D}, \tilde{\mathbf{R}} \mid T, \mathbf{Y}, \phi, \boldsymbol{\mu}, \boldsymbol{\sigma}, \theta). \tag{18}$$

Using the law of conditional probability, we get

$$P(T, \mathbf{Y}, \phi, \boldsymbol{\mu}, \boldsymbol{\sigma} \mid \mathbf{A}, \mathbf{D}, \tilde{\mathbf{R}}, \theta) \propto P(\mathbf{A}, \tilde{\mathbf{R}} \mid \mathbf{D}, \mathbf{Y}, \phi, \boldsymbol{\mu}, \boldsymbol{\sigma}, \theta) P(\mathbf{D}). \tag{19}$$

We have that $P(\mathbf{D})$ is uniform, and after simplification, yields

$$\begin{aligned} P(T, \mathbf{Y}, \phi, \boldsymbol{\mu}, \boldsymbol{\sigma} \mid \mathbf{A}, \mathbf{D}, \tilde{\mathbf{R}}, \theta) &\propto P(\mathbf{A}, \tilde{\mathbf{R}} \mid \mathbf{D}, \mathbf{Y}, \phi, \boldsymbol{\mu}, \boldsymbol{\sigma}, \theta) && (20)\\ &= P(\mathbf{A} \mid \mathbf{D}, \mathbf{Y}, \phi, \boldsymbol{\mu}, \boldsymbol{\sigma}, \theta) P(\tilde{\mathbf{R}} \mid \mathbf{D}, \mathbf{Y}, \phi, \boldsymbol{\mu}, \boldsymbol{\sigma}, \theta) && (21)\\ &= P(\mathbf{A} \mid \mathbf{D}, \mathbf{Y}, \phi, \theta) P(\tilde{\mathbf{R}} \mid \phi, \boldsymbol{\mu}, \boldsymbol{\sigma}). && (22) \end{aligned}$$

We now focus on $P(\mathbf{A} \mid \mathbf{D}, \mathbf{Y}, \phi, \theta)$, which equals

$$\begin{aligned} P(\mathbf{A} \mid \mathbf{D}, \mathbf{Y}, \phi, \theta) &= \prod_{j=1}^{k} \prod_{i=1}^{n} \prod_{q=1}^{m} P(a_{iq} \mid d_{iq}, y_{jq}, \phi, \theta)^{\mathbf{1}\{i \in \phi(j)\}} && (23)\\ &= \prod_{j=1}^{k} \prod_{i=1}^{n} \prod_{q=1}^{m} \left[\sum_{x_{jq} \in S \cup \{0\}} P(a_{iq}, x_{jq} \mid d_{iq}, y_{jq}, \phi, \theta) \right]^{\mathbf{1}\{i \in \phi(j)\}} && (24)\\ &= \prod_{j=1}^{k} \prod_{i=1}^{n} \prod_{q=1}^{m} \left[\sum_{x_{jq} \in S \cup \{0\}} P(a_{iq} \mid d_{iq}, x_{jq}, \phi, \theta) P(x_{jq} \mid y_{jq}, \theta) \right]^{\mathbf{1}\{i \in \phi(j)\}}. && (25) \end{aligned}$$

Bringing everything together, we get

$$
\begin{aligned}
&P(T, \mathbf{Y}, \phi, \boldsymbol{\mu}, \boldsymbol{\sigma} \mid \mathbf{A}, \mathbf{D}, \tilde{\mathbf{R}}, \theta) && (26)\\
&\propto P(\mathbf{A} \mid \mathbf{D}, \mathbf{Y}, \phi, \theta) P(\tilde{\mathbf{R}} \mid \phi, \boldsymbol{\mu}, \boldsymbol{\sigma}) && (27)\\
&= \left(\prod_{j=1}^{k} \prod_{i=1}^{n} \prod_{q=1}^{m} \left[\sum_{x_{jq} \in S \cup \{0\}} P(a_{iq} \mid d_{iq}, x_{jq}, \phi, \theta) P(x_{jq} \mid y_{jq}, \theta) \right]^{\mathbf{1}\{i \in \phi(j)\}} \right) \\
&\quad \cdot \left(\prod_{j=1}^{k} \prod_{i=1}^{n} \prod_{p=1}^{\ell} \mathcal{N}(\mu_{jp}, \sigma_{jp}^2)^{\mathbf{1}\{i \in \phi(j)\}} \right). && (28)
\end{aligned}
$$

We now take the logarithm, yielding

$$
\begin{aligned}
&P(T, \mathbf{Y}, \phi, \boldsymbol{\mu}, \boldsymbol{\sigma} \mid \mathbf{A}, \mathbf{D}, \tilde{\mathbf{R}}, \theta) && (29)\\
&\propto \log P(\mathbf{A} \mid \mathbf{D}, \mathbf{Y}, \phi, \theta) P(\tilde{\mathbf{R}} \mid \phi, \boldsymbol{\mu}, \boldsymbol{\sigma}) && (30)\\
&= \log P(\mathbf{A} \mid \mathbf{D}, \mathbf{Y}, \phi, \theta) + \log P(\tilde{\mathbf{R}} \mid \phi, \boldsymbol{\mu}, \boldsymbol{\sigma}) && (31)\\
&= \sum_{j=1}^{k} \sum_{i=1}^{n} \sum_{q=1}^{m} \mathbf{1}\{i \in \phi(j)\} \log \sum_{x_{jq} \in S \cup \{0\}} P(a_{iq} \mid d_{iq}, x_{jq}, \phi, \theta) P(x_{jq} \mid y_{jq}, \theta) \\
&\quad + \sum_{j=1}^{k} \sum_{i=1}^{n} \sum_{p=1}^{\ell} \mathbf{1}\{i \in \phi(j)\} \log \mathcal{N}(\mu_{jp}, \sigma_{jp}^2). && (32)
\end{aligned}
$$

Via the marginalization of latent VAFs $\mathbf{X}$ and taking the MAP estimates of latent variables $\boldsymbol{\mu}$ and $\boldsymbol{\sigma}$, the above expression allows us to approximate the posterior probability $P(T, \mathbf{Y}, \phi \mid \mathbf{A}, \mathbf{D}, \tilde{\mathbf{R}}, c, \alpha)$ of a given clonal tree T, clonal genotypes $\mathbf{Y}$ and cell clustering ϕ.

A.3 Detectability of a clone

We define *observations* $O(N, M)$ for a subset $N \subseteq [n]$ of cells and a subset $M \subseteq [m]$ of SNVs as

$$
O(N, M) = \sum_{i \in N} \sum_{q \in M} \mathbf{1}\{d_{iq} > 0\}. \tag{33}
$$

Thus, $O(i, M)$ is the number of SNV loci in M that have mapped reads in cell i. Similarly, $O(N, q)$ is the number of cells in N that have mapped reads for SNV locus q. Given a threshold parameter $t \in \mathbb{N}$, we use this function to define when a clone comprised of cells N and newly introduced SNVs M is detectable in the following way.

Definition 1. Given parameter $t \in \mathbb{N}$, a clone comprised of cells N and newly introduced SNVs M is *detectable* provided (i) $\mathrm{median}\,\{O(i, M) \mid i \in N\} \geq t$ and (ii) $\mathrm{median}\,\{O(N, q) \mid q \in M\} \geq t$.

We refer to Fig. B for an example.

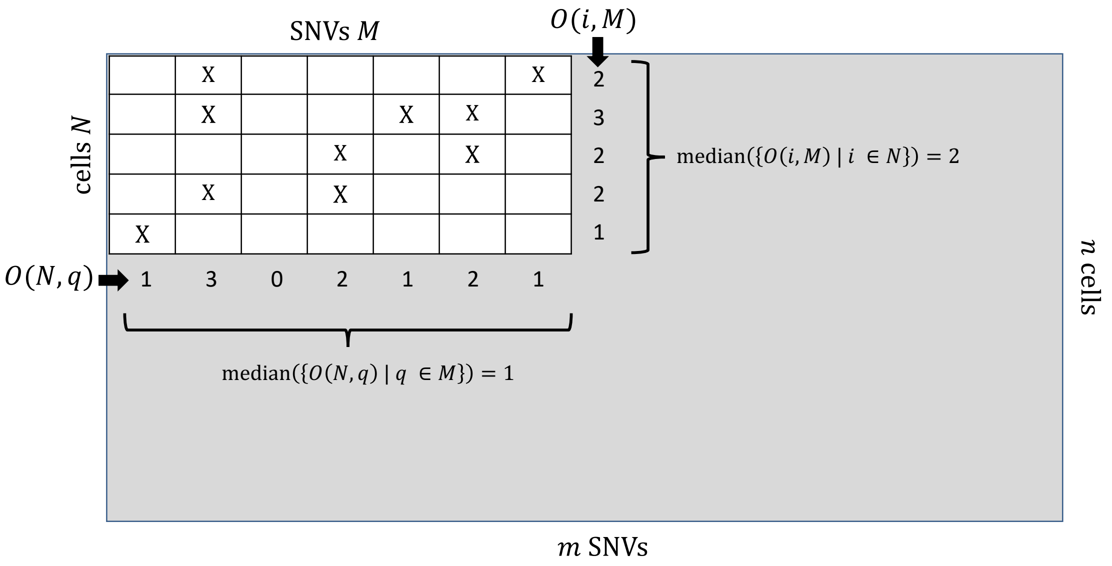

Fig B: **Visual depiction of the detectability of a clone with subset $N \subseteq [n]$ of cells and subset $M \subseteq [m]$ of SNVs.** Here, 'X' indicates the presence of a mapped read for SNV locus q in cell i. Observations $O(N, q)$ is the marginal sum of observations over cells N for each SNV $q \in M$. Similarly, $O(i, M)$ is the marginal sum of observations over SNVs M for each cell $i \in N$. For a clone to be detectable, the medians of these marginal sums must be at least a specified threshold $t \in \mathbb{N}$ (Definition 1).

A.4 PHERTILIZER

PHERTILIZER maintains a set $\mathcal{T}$ of candidate trees throughout three phases: (i) initialization, (ii) growing, and (iii) ranking each tree in $\mathcal{T}$ by its posterior probability. First, in the initialization phase, the set $\mathcal{T}$ is initialized with a single tree containing only a root node v_1. All n cells are assigned to the node's cell cluster $\phi(v_1)$ and all genotypes are initialized to $y_{v_1,q} = 1$ for each SNV q.

Second, in the growing phase (Appendix A.4.1), PHERTILIZER recursively constructs the candidate set $\mathcal{T}$ of clonal trees and the respective clonal genotypes and cell clusterings by performing three different elementary tree operations (`Linear`, `Branching` and `Identity`) on each leaf node v_j of each candidate tree $T \in \mathcal{T}$(Main Text Fig. 2, Fig. D and Fig. E).

The input to each elementary tree operation is a clonal tree T with genotypes $\mathbf{Y}$, cell clustering ϕ and a leaf node v_j. The output is a new clonal tree T' with new genotypes $\mathbf{Y}'$ and cell clustering ϕ'. To define these elementary tree operations more formally, we introduce some notations. Given node v_j not equal to the root v_1, the function $\mathrm{par}(j)$ returns its parent node $v_{\mathrm{par}(j)}$ in clonal tree T. Recall that $\mathbf{y}_j \in \{0,1\}^m$ is the clonal genotype of clone j corresponding to node v_j in T. With slight overloading of notation, $\mathbf{y}_j$ also indicates the set of SNVs present in clone j. We denote the set of SNVs introduced on the incoming edge $(v_{\mathrm{par}(j)}, v_j)$ of node v_j by $\Delta(\mathbf{y}_j) = \mathbf{y}_j \setminus \mathbf{y}_{\mathrm{par}(j)}$.

A `Linear` operation transforms node v_j of clonal tree T with cells $\phi(j)$ and mutations $\Delta(\mathbf{y}_j)$ into a clonal tree T' with a new node v_a and directed edge (v_j, v_a), partitioning original cells $\phi(j)$ and mutations $\Delta(\mathbf{y}_j)$ among nodes v_j and v_a such that the resulting clonal genotypes $\mathbf{y}'_j, \mathbf{y}'_a$ adhere to the infinite sites model, i.e., $\mathbf{y}'_j \subseteq \mathbf{y}'_a$ and $\mathbf{y}'_j \cup \mathbf{y}'_a = \Delta(\mathbf{y}_j)$ (Main Text Fig. 2). Similarly, a `Branching` operation results in a clonal tree T' with two new nodes v_a, v_b that are children of v_j such that the original cells $\phi(j)$ are partitioned between child nodes v_a, v_b, the mutations $\Delta(\mathbf{y}_j)$ are partitioned between all three nodes v_j, v_a, v_b, and the genotypes $\mathbf{y}'_a, \mathbf{y}'_b, \mathbf{y}'_c$ also adhere to the infinite sites model, i.e., $\mathbf{y}'_j \subseteq \mathbf{y}'_a$, $\mathbf{y}'_j \subseteq \mathbf{y}'_b$, $\Delta(\mathbf{y}'_a) \cap \Delta(\mathbf{y}'_b) = \emptyset$ and $\mathbf{y}'_j \cup \mathbf{y}'_a \cup \mathbf{y}'_b = \Delta(\mathbf{y}_j)$. We note that this operation does not assign cells to v_j, thus modeling an extinct clone with SNVs that are common to its children but absent from its parent (Main Text Fig. 2). Lastly, an `Identity` operation on v_j does not alter the tree and prevents the application of other elementary operations on that node (Main Text Fig. 2).

Performing an elementary operation on a clonal tree T with leaf node v_j is equivalent to solving a constrained CTI problem. That is, we seek to infer a subtree rooted at node v_j with maximum posterior probability that is constrained to either be a two node linear subtree or a three node branching subtree. Both operations use coordinate descent to alternately find a partition of cells and SNVs that maximizes the posterior probability of the inferred clonal subtree. For ease of exposition, we describe the input to these operations as a set $N = \phi(j)$ of cells assigned to clone j in tree T and a set $M = \Delta(v_j)$ of SNVs gained on the incoming edge to node v_j. Next, we describe in more detail (i) the growing phase (Appendix A.4.1), (ii) the `Linear` elementary operation (Appendix A.4.2), (iii) the `Branching` elementary operation (Appendix A.4.3), (iv) the `Identity` (Appendix A.4.4) operation, (v) the running time of one elementary tree operation (Appendix A.4.5), (vi) a number of regularization steps to avoid overfitting the data (Appendix A.4.6) and (vii) a post-processing phase to improve clonal genotyping and cell clustering in the inferred clonal tree (Appendix A.4.7).

A.4.1 Growing phase

Elementary tree operations are the building of blocks of clonal trees. Starting from a single tree that contains a single clone containing all SNVs and cells, we consider all possible clonal trees that can be generated via a sequence of elementary tree operations. This is achieved via a growing phase, where all valid candidate clonal trees are generated, or grown, via recursive enumeration.

Specifically, we maintain a candidate set $\mathcal{T}$ of clonal trees. Each candidate tree T is marked as either

visited or unvisited. Whenever a candidate tree is added to the set it is initially marked as unvisited. After a candidate tree is explored, it is marked as visited. To visit a candidate tree $T \in \mathcal{T}$ (Fig. Ca), we consider the subset $L'(T) \subseteq L(T)$ of leaf nodes such that each node $v \in L'(T)$ is not marked as terminal. A leaf node $v \in L(T)$ is marked as terminal if an `Identity` operation was previously applied to the leaf node v. We then apply valid `Linear`, `Branching` and `Identity` operations to each non-terminal leaf node $v \in L'(T)$, storing the extension of the tree (Fig. Cb) to facilitate re-use in other operations. Lastly, we generate new candidate trees to add to the set $\mathcal{T}$ of candidates by applying at most $3^{|L'(T)|} - 1$ different sequences of operations, excluding invalid extensions of a leaf node and the simultaneous application of the identity operation to all leaf nodes (Fig. Cc). Each of these newly generated clonal trees are added to the candidate set $\mathcal{T}$ and marked as unvisited. Candidate tree T is now marked as visited. The growing phase is complete when every candidate tree $T \in \mathcal{T}$ is marked visited.

Although the size of the candidate set grows exponentially, in practice we find that the size of the candidate set is manageable due to the sparsity of data and regularization (Appendix A.4.6). A node tends to become a terminal leaf node after only a few repeated applications of elementary tree operations as one or both of the resulting clones will be undetectable or result in a poor quality extension. This is attributed to either an insufficient number of cells and SNVs that results in the clone failing below the detectablity threshold or because the sequence of prior of elementary operations is such a poor fit to the data that it fails to pass the quality of extension check. These regularization steps (Appendix A.4.6) are key to maintaining a manageable candidate set of clonal trees.

Once the growing phase is complete, we compute the posterior probability $P(T, \mathbf{Y}, \phi \mid \mathbf{A}, \mathbf{D}, \tilde{\mathbf{R}}, c, \alpha)$ of each clonal tree T and the corresponding clonal genotypes $\mathbf{Y}$ and cell clustering ϕ in the candidate set $\mathcal{T}$ in order to rank and prioritize the candidate clonal trees. We return the clonal tree with maximum posterior probability.

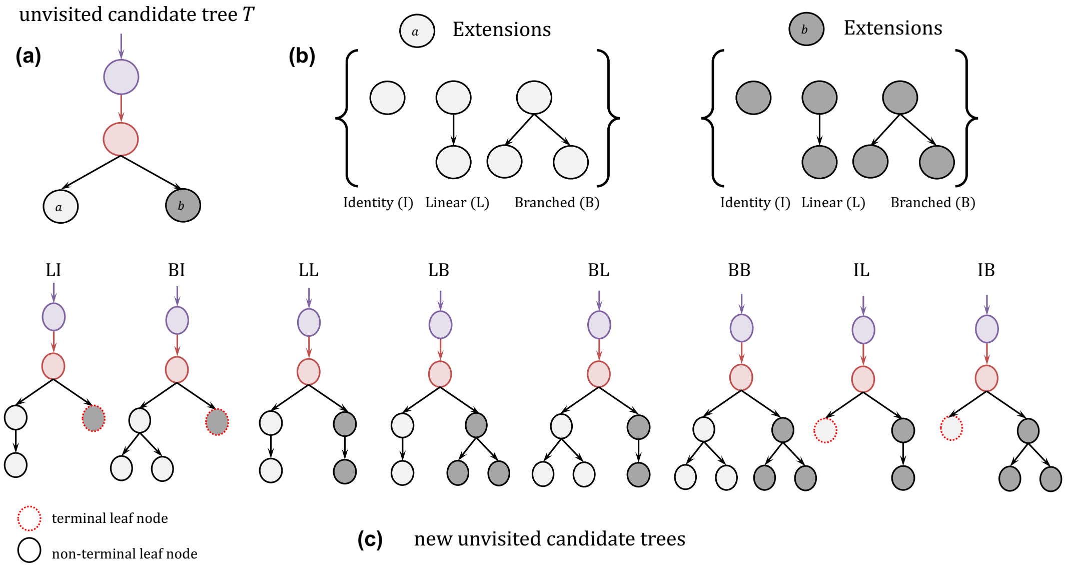

Fig C: **A graphical depiction of PHERTILIZER's recursive enumeration tree building process.** (a) An unvisited candidate clonal tree T in the candidate set $\mathcal{T}$ with two non-terminal leaf nodes $L'(T) = \{a, b\}$. (b) Valid tree extensions of non-terminal leaf nodes $L'(T)$ after application of each of the elementary tree operations. (c) Enumeration of new candidate clonal trees generated by applying each of the 8 valid sequences of elementary operations, i.e., $\{LI, BI, LL, LB, BL, BB, IL, IB\}$, to candidate tree T. These trees are then added to the candidate set $\mathcal{T}$ and marked unvisited. Candidate tree T is marked as visited. This process is repeated until all candidate trees $T \in \mathcal{T}$ are marked visited.

A.4.2 `Linear`

For a given clonal tree T, we specify leaf node v_j as the node on which the `Linear` operation is to be performed. The `Linear` operation (Fig. D) extends tree T to create tree T' by replacing node v_j of tree T by a subtree rooted at node v_j with a single child v_a. Briefly, the `Linear` operation uses a coordinate descent approach to alternately optimize a two-part cell partition and a two-part SNV partition. To optimize the cell partition for a fixed SNV partition, we use the normalized cut algorithm [8]. To optimize the SNV partition for a fixed cell partition, we use our generative model to assign each SNV to the part that maximizes the posterior probability of the extended tree. Next, we describe these steps more formally.

Let $N = \phi(j) \subseteq [n]$ be the cells currently assigned to clone j associated with node v_j and let $M = \Delta(\mathbf{y}_j) \subseteq [m]$ be the set of SNVs introduced on the incoming edge to node v_j of tree T. The output of a `Linear` operation is a two-part partition of the cells N into $\{N_{j'}, N_a\}$ and a two-part partition of the SNVs M into $\{M_{j'}, M_a\}$ (Fig. D). Parts $N_{j'}$ are the cells associated with node v_j in extended tree T' and N_a are the cells associated with the newly added child clone of node v_j in extended tree T'. The part $M_{j'}$ contains SNVs that are introduced on the incoming edge to node v_j in extended tree T' and the part M_a correspond to the SNVs newly introduced on the the incoming edge of the child node of v_j after the completion of the operation.

First, we initialize a `Linear` operation by partitioning uniformly at random the set M of SNVs into two parts $\{M_{j'}, M_a\}$. Given the SNV partition $\{M_{j'}, M_a\}$, we use the normalized cut algorithm [8] to find a two part cell partition $\{N_{j'}, N_a\}$ of cells N. The input to the normalized cut algorithm is a weighed adjacency matrix $\mathbf{W}$, where $w_{ii'}$ relates the similarity of two cells i and i' in the input set. We set the weight $w_{ii'}$ to incorporate both an SNV feature $\mathbf{f}$ and a CNA feature represented by the binned read count embedding $\tilde{\mathbf{R}}$.

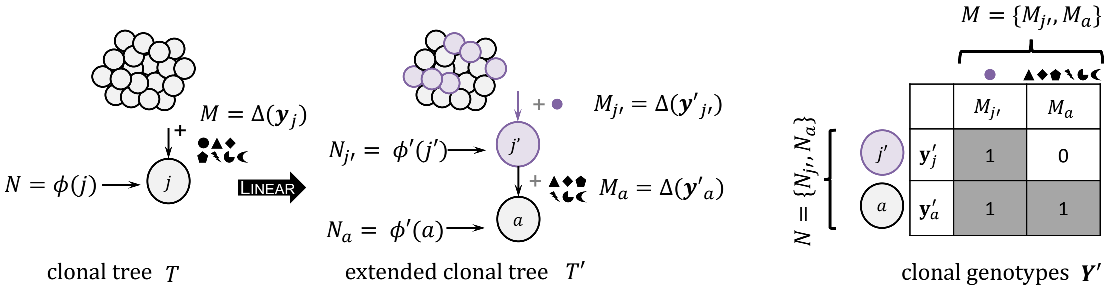

Fig D: **A graphical depiction of a `Linear` elementary tree operation.** The cells $N = \phi(j)$ and the SNVs $M = \Delta(\mathbf{y}_j)$ associated with clone j of tree T are partitioned into $N = \{N_{j'}, N_a\}$ and $M = \{M_{j'}, M_a\}$, respectively. These partitions are then used to update clonal genotypes $\mathbf{Y}'$ and cell clustering ϕ' associated with extended tree T'.

The SNV feature f_i is defined as $f_i = \frac{\sum_{q \in M_a} \mathbf{1}\{a_{iq} > 0\}}{\sum_{q \in M_a} \mathbf{1}\{d_{iq} > 0\}}$. Intuitively, f_i measures for each cell i the proportion of introduced SNV loci M_a with mapped reads for which we observe at least one variant read. In case the denominator is 0 for a cell i, we set $f_i := f_{i''}$ where i'' is the closest cell to i in $\tilde{\mathbf{R}}$ with a nonzero denominator (using Euclidean distance). Then the weight $w_{ii'}$ between cell i and i' is defind as

$$w_{ii'} = e^{\frac{-\|f_i - f_{i'}\|_2^2}{s_f}} \cdot \begin{cases} e^{\frac{-\|\tilde{\mathbf{r}}_i - \tilde{\mathbf{r}}_{i'}\|_2^2}{s_c}}, & \text{if } \|\tilde{\mathbf{r}}_i - \tilde{\mathbf{r}_{i'}}\|_2^2 < \tau, \\ 0, & \text{otherwise,} \end{cases} \tag{34}$$

where τ is a user-specified parameter. We find that in practice, setting τ to the maximum $\|\tilde{\mathbf{r}}_i - \tilde{\mathbf{r}}_{i'}\|_2^2$ over all pairs of cells $i, i' \in N$ works well. However, setting τ more aggressively, i.e., lower, would put greater emphasis on the CNA features and result in a sparser input to the normalized cut algorithm. Following Shi and Malik [8], parameters s_f and s_c are set to 20% of the total range of the $\mathbf{L}^2$ norm for SNV and CNA features, respectively. We use the `scikit-learn` [9] spectral clustering implementation as it solves the normalized cut problem when the number of clusters is 2. We choose the `cluster-qr` [10] strategy to partition cells in the spectral embedding space.

Then, given the updated cell partition $\{N_{j'}, N_a\}$, we optimize the SNV partition $\{M_{j'}, M_a\}$. Let $\mathbf{a}'_{\cdot q}$ and $\mathbf{d}'_{\cdot q}$ be the column vector of variant and total read counts, respectively, corresponding to cells $N_{j'}$. For each SNV $q \in M$, we compute the log-likelihood $\log P(\mathbf{a}'_{\cdot q} \mid \mathbf{d}'_{\cdot q}, N_{j'}, q \in M', \theta)$ where $M' \in \{M_{j'}, M_a\}$. These probabilities are computed as follows.

$$\log P(\mathbf{a}'_{\cdot q} \mid \mathbf{d}'_{\cdot q}, N_{j'}, q \in M_{j'}, \theta) = \sum_{i \in N_{j'}} \log P(a'_{iq} \mid d'_{iq}, y_{j'q} = 1, \theta), \tag{35}$$

$$\log P(\mathbf{a}'_{\cdot q} \mid \mathbf{d}'_{\cdot q}, N_{j'}, q \in M_a, \theta) = \sum_{i \in N_{j'}} \log P(a'_{iq} \mid d'_{iq}, y_{j'q} = 0, \theta). \tag{36}$$

In other words, we compare the probabilities of assigning SNV q to either $M_{j'}$ or M_a. We assign q to the part $M' \in \{M_{j'}, M_a\}$ with maximum log-likelihood. We then iterate until either the cell clusters are not altered from the previous iteration or a user-specified maximum number of iterations is reached (default 50 iterations).

Due to the random initialization of the SNV partition, we repeat this process for a number of restarts. After a restart terminates, we compute a normalized log likelihood term for only the clonal genotypes that will not change even if the leaf nodes of the output tree T' are later extended. Among all restarts, the

`Linear` operation stores the extended tree T', clonal genotypes $\mathbf{Y}'$ and cell clustering ϕ' with maximum normalized log likelihood. For our simulation study, we found that 16 restarts led to stable results. For experimental data, we conservatively used 25 restarts.

Next, we recurse on an input with cells $N := N_{j'}$ and SNVs $M := M_{j'}$. The recursion terminates when either of the newly generated clones fails a regularization check,i.e., a detectability check or quality assessment of an extension. See Appendix A.4.6 for details on these criteria. The recursion step is necessary because the normalized cut algorithm is a recursive partitioning algorithm. Unlike `Branching` (Appendix A.4.3), where all the partitioned cells are associated with leaf nodes in the extended tree nodes, the `Linear` updates the cell cluster of an internal node in the extended tree T'. Since these cells will never be taken as input in any further elementary tree operation, recursions helps to refine the clones in the extended tree T' as much as possible. After the recursion terminates, the `Linear` operation returns the extended tree T', clonal genotypes $\mathbf{Y}'$ and cell clustering ϕ' with maximum normalized log-likelihood.

A.4.3 `Branching`

For a given clonal tree T, we specify leaf node v_j as the node on which the `Branching` operation is to be performed. The `Branching` operation (Fig. E) extends tree T to create tree T' by replacing node v_j of tree T by a binary subtree rooted at node v_j with two children v_a and v_b. Briefly, the `Branching` operation uses a coordinate descent approach to alternately optimize a two-part cell partition and a three-part SNV partition. To optimize the cell partition for a fixed SNV partition, we use the normalized cut algorithm [8].

To optimize the SNV partition for a fixed cell partition, we use our generative model to assign each SNV to the part that maximizes the posterior probability of the extended tree. Next, we describe these steps more formally.

Let $N = \phi(j) \subseteq [n]$ be the cells currently assigned to clone j associated with node v_j and let $M = \Delta(\mathbf{y}_j) \subseteq [m]$ be the set of SNVs introduced on the incoming edge to node v_j for tree T. The output of a `Branching` operation is a two-part partition of the cells N into $\{N_a, N_b\}$ and a partition of SNVs M into three parts $\{M_{j'}, M_a, M_b\}$ (Fig. E). Parts N_a and N_b are the cells assigned to the two clones associated with the newly added two children of node v_j in extended tree T'. The part $M_{j'}$ contains SNVs that are introduced on the incoming edge to node v_j in extended tree T'. Parts M_a and M_b correspond to the SNVs newly introduced on the two incoming edges of the two children of node v_j after the completion of the operation.

First, we initialize a `Branching` operation by partitioning uniformly at random the set M of SNVs into three parts $\{M_{j'}, M_a, M_b\}$. Similarly to `Linear` (Appendix A.4.2), we use the SNV partition to find a two part cell partition $\{N_a, N_b\}$ of cells N.

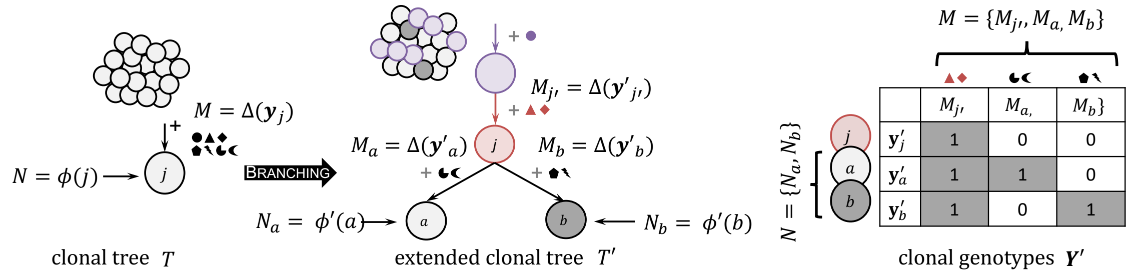

Fig E: **A graphical depiction of a `Branching` elementary tree operation.** The cells $N = \phi(j)$ and the SNVs $M = \Delta(\mathbf{y}_j)$ associated with clone j of tree T are partitioned into $N = \{N_a, N_b\}$ and $M = \{M_{j'}, M_a, M_b\}$, respectively. These partitions are then used to update clonal genotypes $\mathbf{Y}'$ and cell clustering ϕ' associated with extended tree T'.

The SNV feature is set as $\mathbf{f}_i = [f_{ia}, f_{ib}]^\top$, where

$$f_{ia} = \frac{\sum_{q \in M_a} \mathbf{1}\{a_{iq} > 0\}}{\sum_{q \in M_a} \mathbf{1}\{d_{iq} > 0\}}. \tag{37}$$

In words, f_{ia} measures for each cell i the proportion of SNVs in set M_ℓ with mapped reads for which we observe at least one variant read. In case the denominator is 0 for a cell i, we set $f_{ia} := f_{i''a}$ where i'' is the closest cell to i in $\tilde{\mathbf{R}}$ with a nonzero denominator (using Euclidean distance). SNV feature f_{ib} is similarly defined for SNVs M_b.

$$w_{ii'} = e^{\frac{-\|\mathbf{f}_i - \mathbf{f}_{i'}\|_2^2}{s_f}} \cdot \begin{cases} e^{\frac{-\|\tilde{\mathbf{r}}_i - \tilde{\mathbf{r}}_{i'}\|_2^2}{s_c}}, & \text{if } \|\tilde{\mathbf{r}}_i - \tilde{\mathbf{r}_{i'}}\|_2^2 < \tau, \\ 0, & \text{otherwise}, \end{cases} \tag{38}$$

where τ is a user-specified parameter. We find that in practice, setting τ to the maximum $\|\tilde{\mathbf{r}}_i - \tilde{\mathbf{r}}_{i'}\|_2^2$ over all pairs of cells $i, i' \in N$ works well. However, setting τ more aggressively, i.e., lower, would put greater emphasis on the CNA features and result in a sparser input to the normalized cut algorithm. Following Shi and Malik [8], parameters s_f and s_c are set to 20% of the total range of the $\mathbf{L}^2$ norm for SNV and CNA features, respectively. We use the `scikit-learn` [9] spectral clustering implementation as it solves the normalized cut problem when the number of clusters is 2. We choose the `cluster-qr` [10] strategy to partition cells in the spectral embedding space.

Then, given the updated cell partition $\{N_a, N_b\}$, we optimize the SNV partition $\{M_{j'}, M_a, M_b\}$. Let $\mathbf{a}'_{\cdot q}$ and $\mathbf{d}'_{\cdot q}$ be the column vector of variant and total read counts, respectively, corresponding to cells N. For each SNV $q \in M$, we compute the log-likelihood $\log P(\mathbf{a}'_{\cdot q} \mid \mathbf{d}'_{\cdot q}, N = \{N_a, N_b\}, q \in M', \theta)$ where $M' \in \{M_{j'}, M_a, M_b\}$. These probabilities are computed as follows.

$$\log P(\mathbf{a}'_{\cdot q} \mid \mathbf{d}'_{\cdot q}, N = \{N_a, N_b\}, q \in M_{j'}, \theta) = \sum_{i \in N} \log P(a'_{iq} \mid d'_{iq}, y_{j'q} = 1, \theta), \tag{39}$$

$$\begin{aligned} \log P(\mathbf{a}'_{\cdot q} \mid \mathbf{d}'_{\cdot q}, N = \{N_a, N_b\}, q \in M_a, \theta) = &\sum_{i \in N_a} \log P(a'_{iq} \mid d'_{iq}, y_{aq} = 1, \theta) \\ &+ \sum_{i \in N_b} \log P(a'_{iq} \mid d'_{iq}, y_{bq} = 0, \theta), \end{aligned} \tag{40}$$

$$\begin{aligned} \log P(\mathbf{a}'_{\cdot q} \mid \mathbf{d}'_{\cdot q}, N = \{N_a, N_b\}, q \in M_b, \theta) = &\sum_{i \in N_b} \log P(a'_{iq} \mid d'_{iq}, y_{bq} = 1, \theta) \\ &+ \sum_{i \in N_a} \log P(a'_{iq} \mid d'_{iq}, y_{aq} = 0, \theta). \end{aligned} \tag{41}$$

In other words, we compare the probabilities of assigning SNV q to either $M_{j'}$, M_a or M_a. We assign q to the part $M' \in \{M_{j'}, M_a, M_b\}$ with maximum log-likelihood. We then iterate until either the cell clusters are not altered from the previous iteration or a user-specified maximum number of iterations is reached.

Due to the random initialization of the SNV partition, we repeat this process for a number of restarts. After a restart terminates, we compute a normalized log likelihood term for only the clonal genotypes that will not change even if the leaf nodes of the output tree T' are later extended. Among all restarts, the `Branching` operation returns the extended tree T', clonal genotypes $\mathbf{Y}'$ and cell clustering ϕ' with maximum normalized log likelihood. For our simulation study, we found that 16 restarts led to stable results. For experimental data, we conservatively used 25 restarts.

A.4.4 `Identity`

An `Identity` elementary operation is a trivial operation that simply returns an unmodified clonal tree and marks the inputted leaf node as terminal. For PHERTILIZER, application of the `Identity` operation is most useful during the growing, or recursive enumeration, stage (Appendix A.4.1) of the algorithm.

A.4.5 Running time of an elementary tree operation

Both `Linear` and `Branching` elementary tree operations require a cell partitioning step and SNV partitioning step. Thus, the running time of a single elementary tree operation is the combination of these two steps. The running time of cell clustering is $O(n^3)$ as it involves an eigenvalue decomposition [8]. In practice, we use the spectral clustering algorithm included in scikit-learn [9], which for two clusters reduces to the normalized cut algorithm. To partition SNVs, we pre-compute the log-likelihood $\log P(a_{iq} \mid d_{iq}, y = \ell, \theta)$ for each $\ell \in \{0, 1\}$,for each cell $i \in [n]$ and for each SNV $j \in [m]$ at the start of the algorithm. Given these log-likelihood values and a cell partition, we can partition SNVs in $O(nm)$. Thus, the total running time of a elementary tree operation is $O(n^3 + nm)$.

A.4.6 Regularization

To avoid overfitting the input data, we execute a number of regularization steps after performing an elementary tree operation on a node v_j of clonal tree T.

Detectability check. We assess whether the clones associated with new nodes v_j, v_a, v_b for `Branching` (Appendix A.4.3) and nodes v_j, v_a for `Linear` (Appendix A.4.2) are detectable (Appendix A.3). We set the default as $t = 4$.

Quality assessment of an extension. Even though we may have enough data observations to theoretically perform an elementary operation on a leaf node v_j in tree T, a high quality extension might not exist. We define the *cell mutational burden* (CMB) for a cell $i \in N \subseteq [n]$ and set $M \subseteq [m]$ of SNVs as

$$\mathrm{CMB}(i, M) = \frac{\sum_{q \in M} \mathbf{1}\{a_{iq} > 0\}}{\sum_{q \in M} \mathbf{1}\{d_{iq} > 0\}}. \tag{42}$$

More details and intuition on the CMB is provided in Appendix B.2.3.

We define a quality check $\mathrm{qc}(N, M)$ as $\mathrm{median}\{\mathrm{CMB}(i, M) \mid i \in [N]\}$. When attempting a `Linear` operation, which partitions $N = \phi(j)$ into parts $\{N_{j'}, N_a\}$ and SNVs $M = \Delta(\mathbf{y}_j)$ into parts $\{M_{j'}, M_a\}$ (Appendix A.4.2) we expect $\mathrm{qc}(N, M_{j'})$ to be high, i.e., at least 0.15, since we expect all $M_{j'}$ SNVs to be present in the set N of cells. Conversely, we expect $\mathrm{qc}(N_{j'}, M_a)$ to be low, i.e., at most 0.05, since we do not expect to see SNVs M_a in the set $N_{j'}$ of cells. For similar reasoning as above, we define the following quality checks for the `Branching` operation (Appendix A.4.3).

$$\mathrm{qc}(N, M_{j'}) \geq 0.15,$$
$$\mathrm{qc}(N_a, M_b) \leq 0.05,$$
$$\mathrm{qc}(N_b, M_a)) \leq 0.05.$$

We use 0.05 and 0.15 for illustrative purposes and because we set these as the default values. However, the user may specify different values for these parameters to be less conservative.

A.4.7 Postprocessing

Recall that the input to an elementary operation is a node v_j with attached cells $\phi(j)$ and introduced SNVs $\Delta(\mathbf{y}_j)$. It may be the case that a cell i in $\phi(j)$ has zero variant reads for all SNVs in $\Delta(\mathbf{y}_j)$, i.e., $a_{iq} = 0$ for all SNVs $q \in \Delta(\mathbf{y}_j)$. Similarly, an SNV q in $\Delta(\mathbf{y}_j)$ may not have any cell in $\phi(j)$ with supporting variant reads, i.e., $a_{iq} = 0$ for all cells $i \in \phi(j)$. These SNVs and cells are uninformative during the elementary operations and their placement within the tree is best determined after the clonal tree has been identified. As such, prior to performing an elementary operation on node v_j, we remove such SNVs and cells, and add them to a set M of SNVs and a set N of cells, respectively. After PHERTILIZER returns the clonal tree with maximum posterior probability, we then perform an optional post-processing phase that contains two stages: (i) the placement of SNVs and cells that were removed from the tree during inference and (ii) the reassignment of SNVs that fit poorly in the tree.

For post-processing, we are given a clonal tree T, clonal genotypes $\mathbf{Y}$ and cell clustering ϕ, as well as the cells N and SNVs M as described above. In the first stage, we first fix the cell clustering ϕ. Recall that $\Delta(\mathbf{y}_j)$ is the set of SNVs introduced on the incoming edge to node v_j in tree T. Then, for each SNV $q \in M$ and each node $v_j \in V(T)$, we compute the likelihood that SNV q is gained at node v_j in tree T as follows.

$$\begin{aligned}\log P(\mathbf{a}_{\cdot q} \mid \mathbf{d}_{\cdot q}, \phi, q \in \Delta(\mathbf{y}_j), T, \theta) = & \sum_{v_{j'} \in \mathrm{desc}(v_j) \cup \{v_j\}} \sum_{i \in \phi(j')} \log P(a_{iq} \mid d_{iq}, y_{j'q} = 1, \theta) \\ & + \sum_{v_{j'} \in \mathrm{anc}(v_j)} \sum_{i \in \phi(j')} \log P(a_{iq} \mid d_{iq}, y_{j'q} = 0, \theta),\end{aligned} \tag{43}$$

where $\mathrm{desc}(v_j)$ is the set of descendant nodes and $\mathrm{anc}(v_j)$ is the set of ancestor nodes of node v_j in tree T. We assign q to the incoming edge of node $v_j \in T$ with maximum log-likelihood, resulting in updated clonal genotypes $\mathbf{Y}'$. We then set clonal genotypes $\mathbf{Y} := \mathbf{Y}'$.

Next, we repeat the above process but fix the clonal genotypes $\mathbf{Y}$ and update the cell clustering ϕ. For each cell $i \in N$ and for each node $v_j \in V(T)$ such that $\phi(j) \neq \emptyset$, we compute the log-likelihood of cell i being assigned to clone j as follows.

$$\begin{aligned}\log P(\mathbf{a}_{\cdot q} \mid \mathbf{d}_{\cdot q}, \mathbf{Y}, i \in \phi(j), T, \theta) = & \sum_{v_{j'} \in \mathrm{anc}(v_j) \cup \{v_j\}} \sum_{q \in \Delta(\mathbf{y}_{j'})} \log P(a_{iq} \mid d_{iq}, y_{j'q} = 1, \theta) \\ & + \sum_{v_{j'} \in \mathrm{desc}(v_j)} \sum_{q \in \Delta(\mathbf{y}_{j'})} \log P(a_{iq} \mid d_{iq}, y_{j'q} = 0, \theta).\end{aligned} \tag{44}$$

We assign cell i to the cell cluster $j \in [k]$, with maximum log-likelihood resulting in updated cell clustering ϕ'. We then set cell clusterings $\phi := \phi'$.

In the second stage, given a tree T and a cell clustering ϕ, we attempt to improve clonal genotypes $\mathbf{Y}$ by moving SNVs that fit poorly with the given cell clustering ϕ and tree T. We iterate over the nodes of tree T using a postorder traversal. At each node v_j, we identify poorly fitting SNVs by computing a metric we refer to as the binary variant alele frequency (BVAF), which is as follows for a given SNV q and node v_j

$$\mathrm{BVAF}(q, v_j) = \frac{\sum_{v_{j'} \in \mathrm{desc}(v_j) \cup \{v_j\}} \sum_{i \in \phi(j')} \mathbf{1}\{a_{iq} > 0\}}{\sum_{v_{j'} \in \mathrm{desc}(v_j) \cup \{v_j\}} \sum_{i \in \phi(j')} \mathbf{1}\{d_{iq} > 0\}}. \tag{45}$$

In words, $\mathrm{BVAF}(q, v_j)$ is the fraction of cells in clade v_j with mapped variant reads at SNV q. We compute $\mathrm{BVAF}(q, v_j)$ for every SNV $q \in \Delta(\mathbf{y}_j)$. We identify a cutoff t' at the 10th percentile of this distribution. Then, any SNV q where $\mathrm{BVAF}(q, v_j) \leq t'$ is removed from the tree and placed in a candidate set M of SNVs to be reassigned. Reassignment then proceeds as described above.

We repeat this second stage of post-processing for either a fixed number of iterations or until no SNVs are reassigned, resulting in updated clonal genotypes $\mathbf{Y}'$. After termination, we update clonal genotypes $\mathbf{Y} := \mathbf{Y}'$ and return clonal genotypes $\mathbf{Y}$ and cell clustering ϕ.

B Supplementary results

B.1 Simulation study

Below we provide pertinent details related to our simulation study and supplemental figures.

- Appendix B.1.1 provides additional details for the BASELINE comparison method
- Appendix B.1.2 provides additional details on our simulation study design
- Appendix B.1.3 provides the specification of runtime parameters for methods analyzed in the simulation study
- Appendix B.1.4 provides additional details on the performance metrics used to evaluation the results of our simulation study
- Appendix B.1.5 contains supplemental results figures for our simulation study

B.1.1 BASELINE method

The BASELINE method seeks to infer clonal genotypes for cells utilizing copy-number information embedded in the binned read counts $\mathbf{R}$ via the following three steps. First, the method projects binned read counts $\mathbf{R} \in \mathbb{R}^{n \times b}$ into a low dimensional space $\tilde{\mathbf{R}} \in \mathbb{R}^{n \times \ell}$ (i.e., $\ell \ll b$) — see Appendix A.1. Second, it clusters cells into clones in the low-dimensional binned read count embedding $\tilde{\mathbf{R}}$. Third, each cluster of cells is treated as a pseudobulk sample and the cluster is genotyped. In our implementation, we used OPTICS [11] for the clustering step, since it is in the family of DBSCAN clustering methods that is a common choice used for the BASELINE in practice and the number of clusters does not need to be specified *a priori*. To genotype a cell cluster j, we set clonal genotype $y_{jq} = 1$ for each SNV $q \in [m]$ whenever the variant allele frequency, i.e., the total number of variant reads/total number of reads, was greater than 0.05 and $y_{jq} = 0$ otherwise. Fig. F shows good concordance of clonal genotypes using this approach with that of the inferred clonal genotypes on a DLP+ dataset [1].

B.1.2 Simulation setup

We generated scDNA-seq data from *in silico* heterogeneous tumors with both CNAs and SNVs. We generated a tree T^* with $k \in \{5, 9\}$ nodes. We varied the number $n \in \{1000, 2000\}$ of sequenced cells and the number $m \in \{5000, 10000, 15000\}$ of SNVs. We generated read counts following a beta-binomial distribution with coverage of $0.01\times$, $0.05\times$, and $0.1\times$. We introduced 3 chromosome-level and 8 chromosome-arm level CNAs, affecting a total of $\ell = 577$ bins each of length 5 MB spanning all autosomes. We sampled a cell clustering ϕ^* as well as SNV and CNA placement on edges from a symmetric Dirichlet distribution with a concentration parameter of 2.

Each combination of simulation parameters was replicated with ten different random number generator seeds, amounting to a total of 180 experiments. In addition, we generated a set of simulation experiments in the same manner as above with exception of fixing the copy number as heterzygous diploid at every locus in the genome. We also varied the SNV evolutionary model, using either a Dollo (SNV loss, [12])

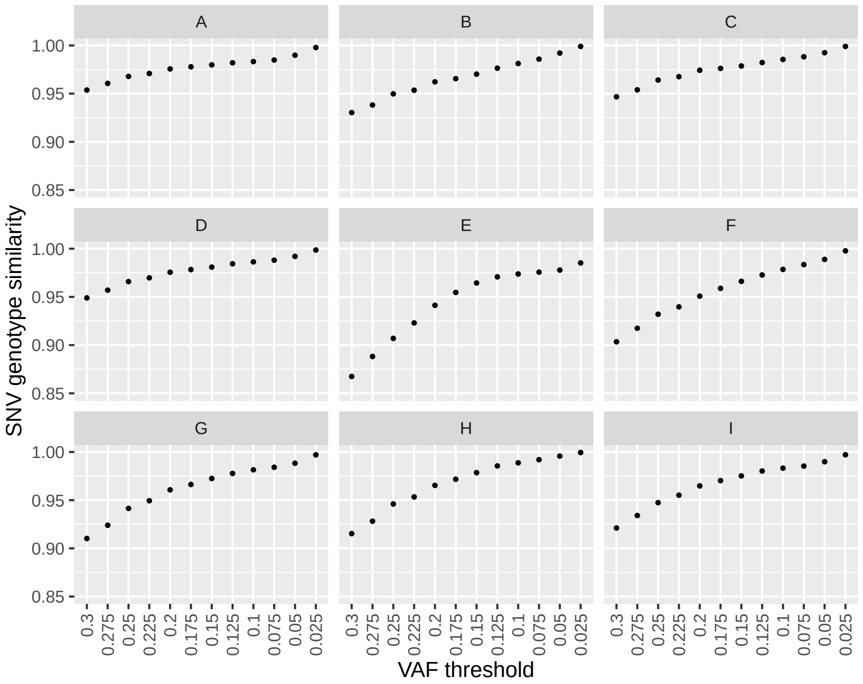

Fig F: **Genotype similarity between inferred genotypes per cell cluster using the BASELINE method and varying the variant allele frequency (VAF) threshold and inferred clonal genotypes per cell cluster by Laks et al. [1]**

or an infinite sites model (without SNV loss, [13]). We used a mean sequencing coverage of $0.01\times$ drawn from a Poisson distribution, to generate data matrices $\mathbf{D}, \mathbf{R}$ and sampled the variant count matrix $\mathbf{A}$ from a binomial distribution parameterized by the ground truth VAF.

B.1.3 Runtime parameters

PHERTILIZER. We used default parameters specified below for all simulation instances.

```
phertilizer -f  ${input_dataframe_tsv}$ \
    --bin_count_data ${input_reads_per_bin_file}$ \
    --radius 1 -a 0.001 \
    --min_obs 7 \
    -c 5 \
    --post_process \
    --use_copy_kernel \
    -d ${seed}$ \
    -j 50 \
    -s 16 \
    --low_cmb 0.05 \
    --high_cmb 0.15 \
    --nobs_per_cluster 3
```

SBMCLONE. We used the same seed for simulation as the seed for SBMClone.

```
python SBMClone/sbmclone.py -o ${out_dir} --seed $SEED \
    ${input_dataframe_tsv}

```

SCITE. We set the maximum MCMC chain length to be $900,000$, repetitions to 3, false positive rate to be 0.02, false negative rate to be 0.01, and seed to 42.

```
scite -i ${input} -n ${mut_clusters} -m ${cell_clusters} \
        -r 3 -l 900000 \
        -fd 0.02 -ad 0.01 \
        -names ${names} \
        -seed 42 \
        -a -o ${outprefix}
```

B.1.4 Performance metrics

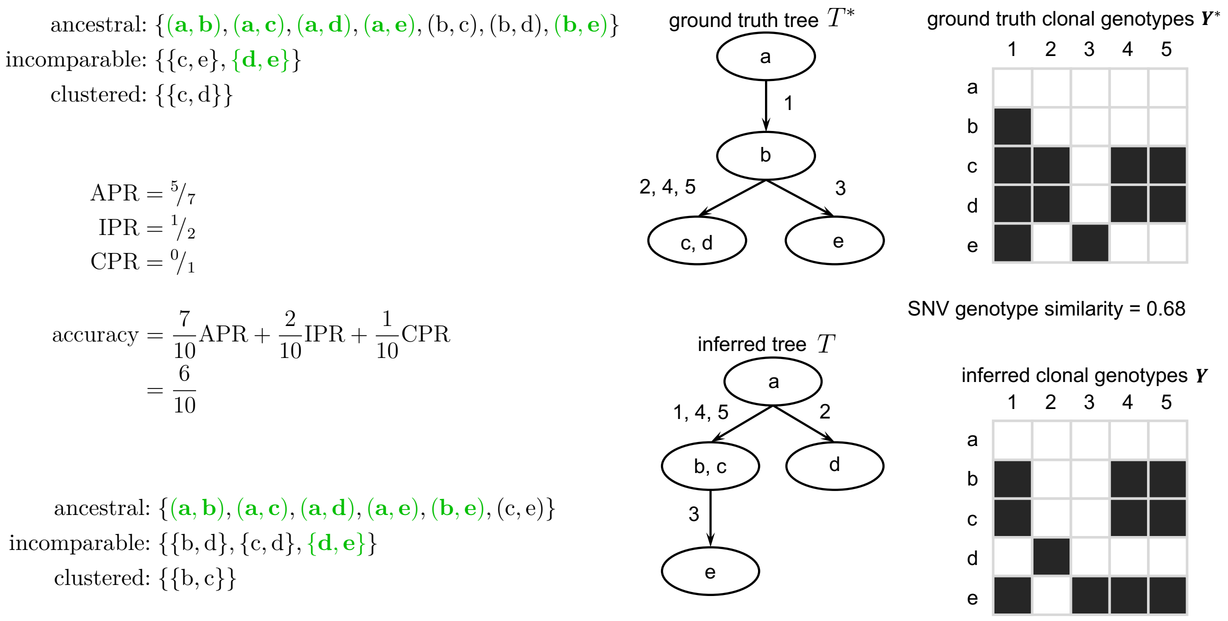

Fig G: **Example for ancestral pair recall (APR), clustered pair recall (CPR), incomparable pair recall (IPR), and accuracy for cells.** To the right of the trees, we show the ground truth and inferred genotypes $\mathbf{Y}^*$ and $\mathbf{Y}$ projected down to individual cells. Main Text Fig. 3 provides an example of APR, CPR, IPR and accuracy metrics for SNVs.

We assessed the quality of an inferred solution $(T, \phi, \mathbf{Y})$ against a ground-truth tree T^*, cell clustering ϕ^* and clonal genotypes $\mathbf{Y}^*$ using ancestral pair recall (APR), incomparable pair recall (IPR), and clustered pair recall (CPR) metrics for cells and SNVs [14], as well as genotype similarity. In addition, we compute a single *accuracy* value ($\in [0, 1]$) composed of the weighted average of APR, IPR and CPR — where the weights are proportional to the number of pairs in each class. We note that an SNV and cell accuracy of 1 implies that the inferred solution perfectly matches ground truth.

APR, IPR, CPR. For any two SNVs $q \neq q'$ there are three possible placements in the tree: (i) *ancestral*: q is gained on a node that is distinct and ancestral to the node where q' is gained; (ii) *clustered*: q and q' are both gained on the same node; and (iii) *incomparable*: q and q' are gained on distinct nodes that occur on distinct branches of the tree. Similarly, two distinct cells $i \neq i'$ have the same three possible placements. The APR assesses the ratio of ancestral pairs from T^* recalled in T, whereas CPR and IPR do so for clustered and incomparable pairs, respectively. Thus, if $\mathrm{APR} = \mathrm{CPR} = \mathrm{IPR} = 1$ for both cells and SNVs then the inferred solution $(T, \phi, \mathbf{Y})$ is identical to the ground truth $(T^*, \phi^*, \mathbf{Y}^*)$. Main Text Fig. 3 and Fig. G provide a graphical depiction of these metrics.

Genotype similarility. We define the *genotype similarity* as 1 minus the normalized Hamming distance between ground truth genotypes and inferred genotypes of cells. More formally, this is defined as $1/(nm) \sum_{i=1}^{n} \sum_{q=1}^{m} \mathbb{1}(y_{\bar{\phi}^*(i),j} = y^*_{\bar{\phi}(i),q})$ where $\bar{\phi}(i)$ ($\bar{\phi}^*(i)$) is the unique clone j such that $i \in \phi(j)$ ($i \in \phi^*(j)$), given an inferred genotype $\hat{\mathbf{y}}_i \in \{0,1\}^m$ and a ground truth genotype $\mathbf{y}^*_i$. Thus, a genotype similarity of 1 implies that every sequenced cell was correctly genotyped. Fig. G above provides a visualization for the computation of this metric.

B.1.5 Supplemental simulation study figures

- Fig. H shows supplemental simulation study results for coverage $0.01\times$
- Fig. I shows supplemental simulation study results for coverage $0.05\times$
- Fig. J shows supplemental simulation study results for coverage $0.1\times$
- Fig. K shows heterozygous diploid simulation study results
- Fig. L shows Dollo evolutionary model simulation study results
- Fig. M shows a running time comparison for the simulation study
- Fig. N shows sensitivity of genotype similarity to the choice of hyperparameters
- Fig. O shows sensitivity of tree reconstruction accuracy to the choice of hyperparameters

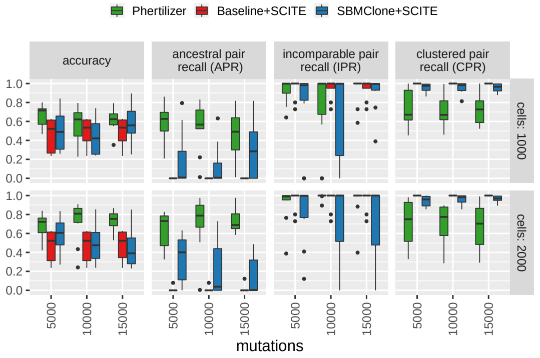

(a) cell placement metrics for $k = 5$ clones

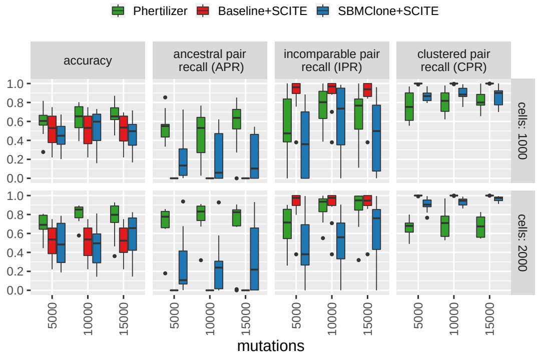

(b) cell placement metrics for $k = 9$ clones

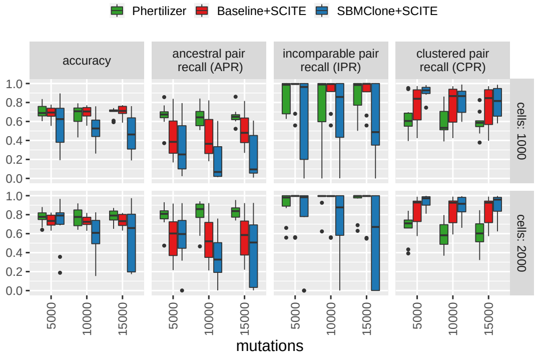

(c) SNV placement metrics for $k = 5$ clones

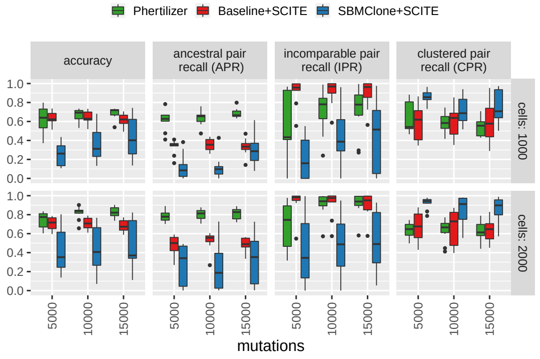

(d) SNV placement metrics for $k = 9$ clones

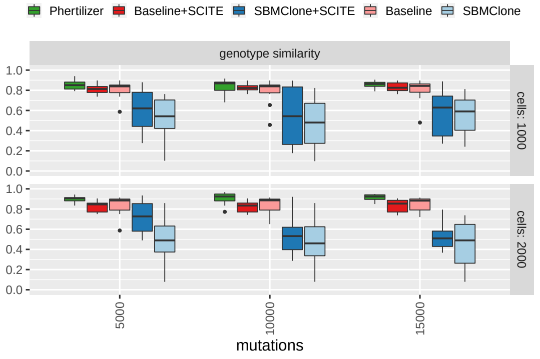

(e) genotype similarity for $k = 5$ clones

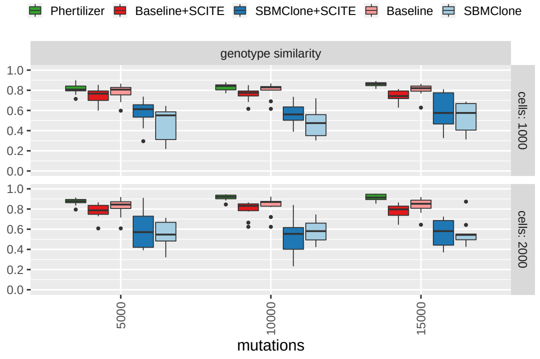

(f) genotype similarity for $k = 9$ clones

Fig H: **Simulation results for coverage** $g = 0.01\times$

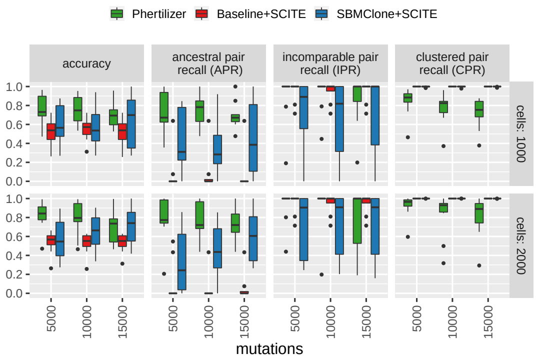

(a) cell placement metrics for $k = 5$ clones

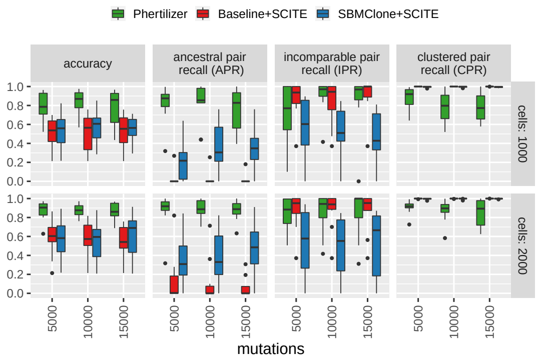

(b) cell placement metrics for $k = 9$ clones

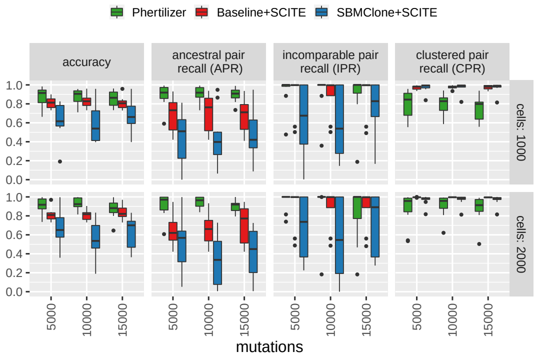

(c) SNV placement metrics for $k = 5$ clones

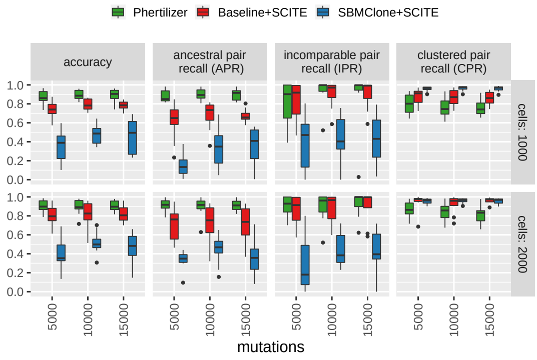

(d) SNV placement metrics for $k = 9$ clones

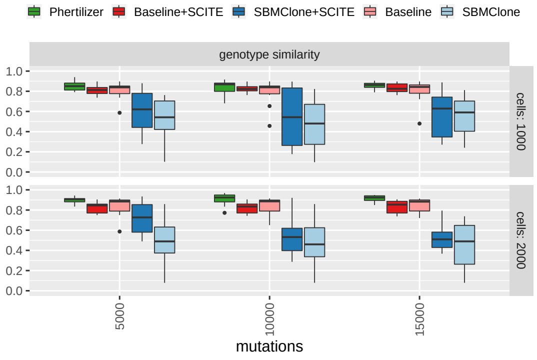

(e) genotype similarity for $k = 5$ clones

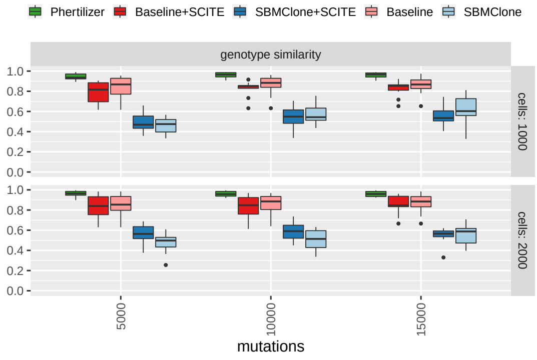

(f) genotype similarity for $k = 9$ clones

Fig I: **Simulation results for coverage $g = 0.05\times$.**

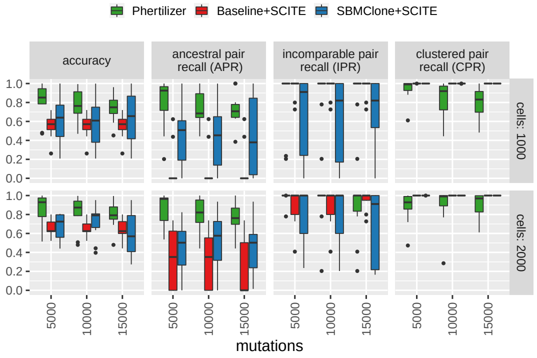

(a) cell placement metrics for $k = 5$ clones

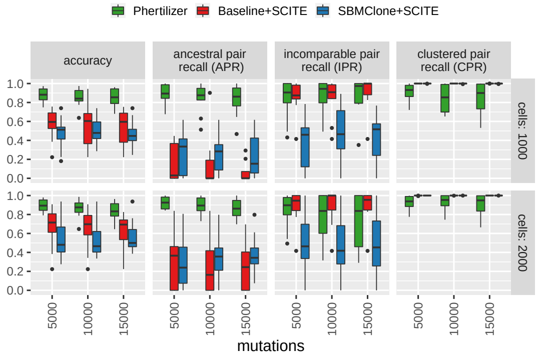

(b) cell placement metrics for $k = 9$ clones

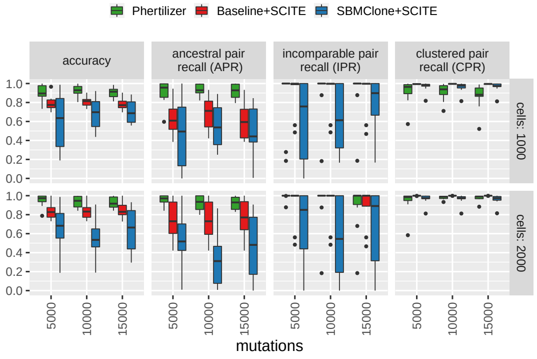

(c) SNV placement metrics for $k = 5$ clones

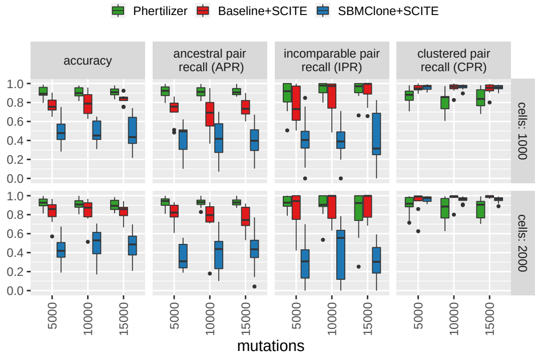

(d) SNV placement metrics for $k = 9$ clones

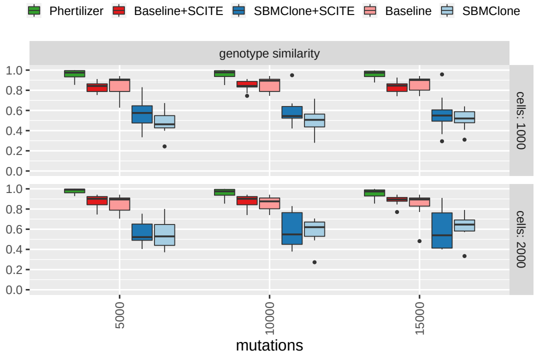

(e) genotype similarity for $k = 5$ clones

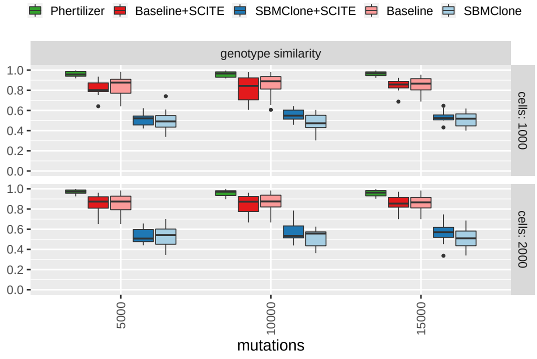

(f) genotype similarity for $k = 9$ clones

Fig J: **Simulation results for coverage $g = 0.1\times$.**

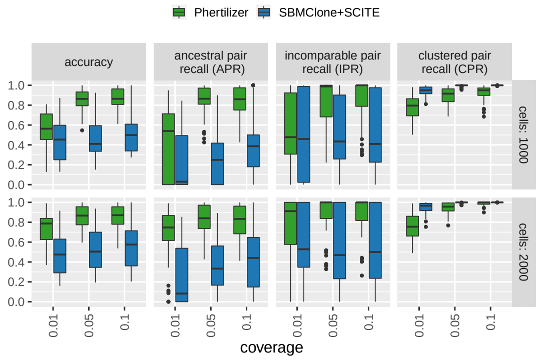

(a) cell placement metrics

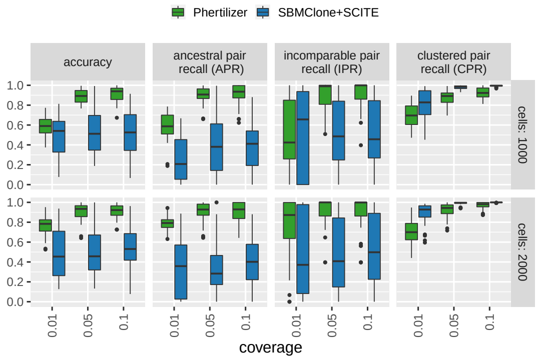

(b) SNV placement metrics

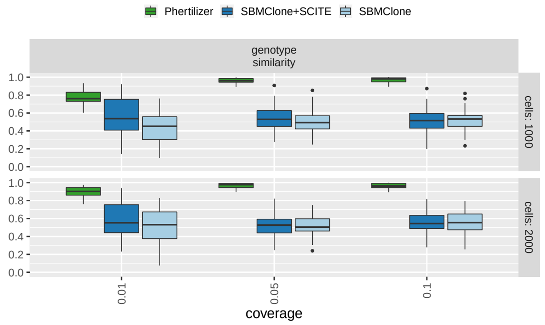

(c) genotype similarity

Fig K: **Heterozygous diploid simulation results aggregrated over $k \in \{5, 9\}$ clones and $m \in \{5000, 10000, 15000\}$ SNVs.** BASELINE+SCITE was excluded from comparison because the lack of CNAs in the data resulted in only a single clone being identified in the read count embedding space for each instance.

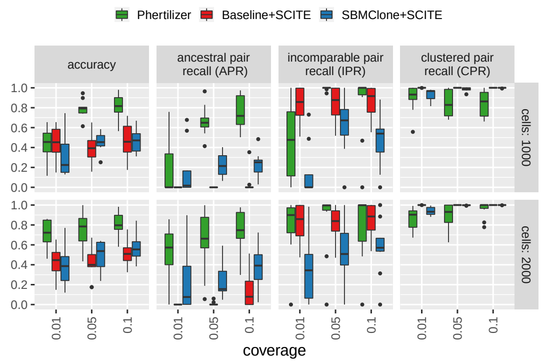

(a) cell placement metrics

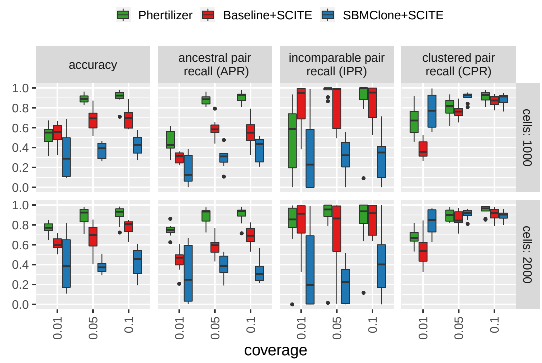

(b) SNV placement metrics

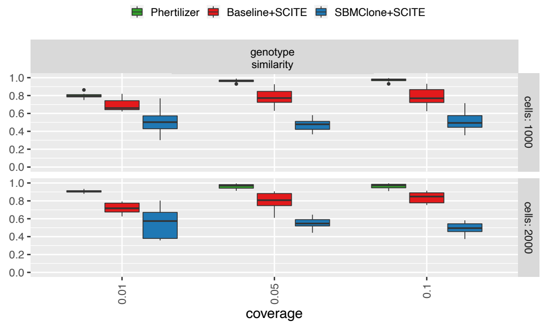

(c) genotype similarity

Fig L: **Dollo evolutionary model simulation results for $k = 9$ clones and $m = 15000$ SNVs**

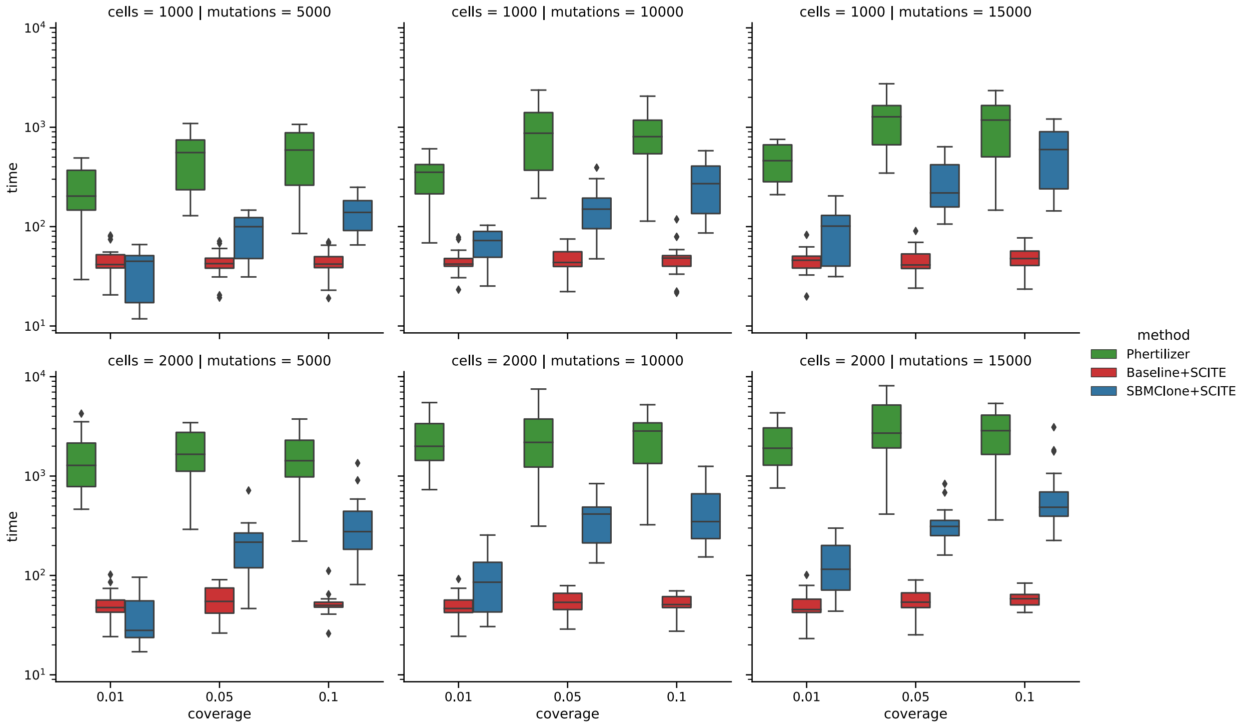

Fig M: **Running time (in seconds) on simulation data**

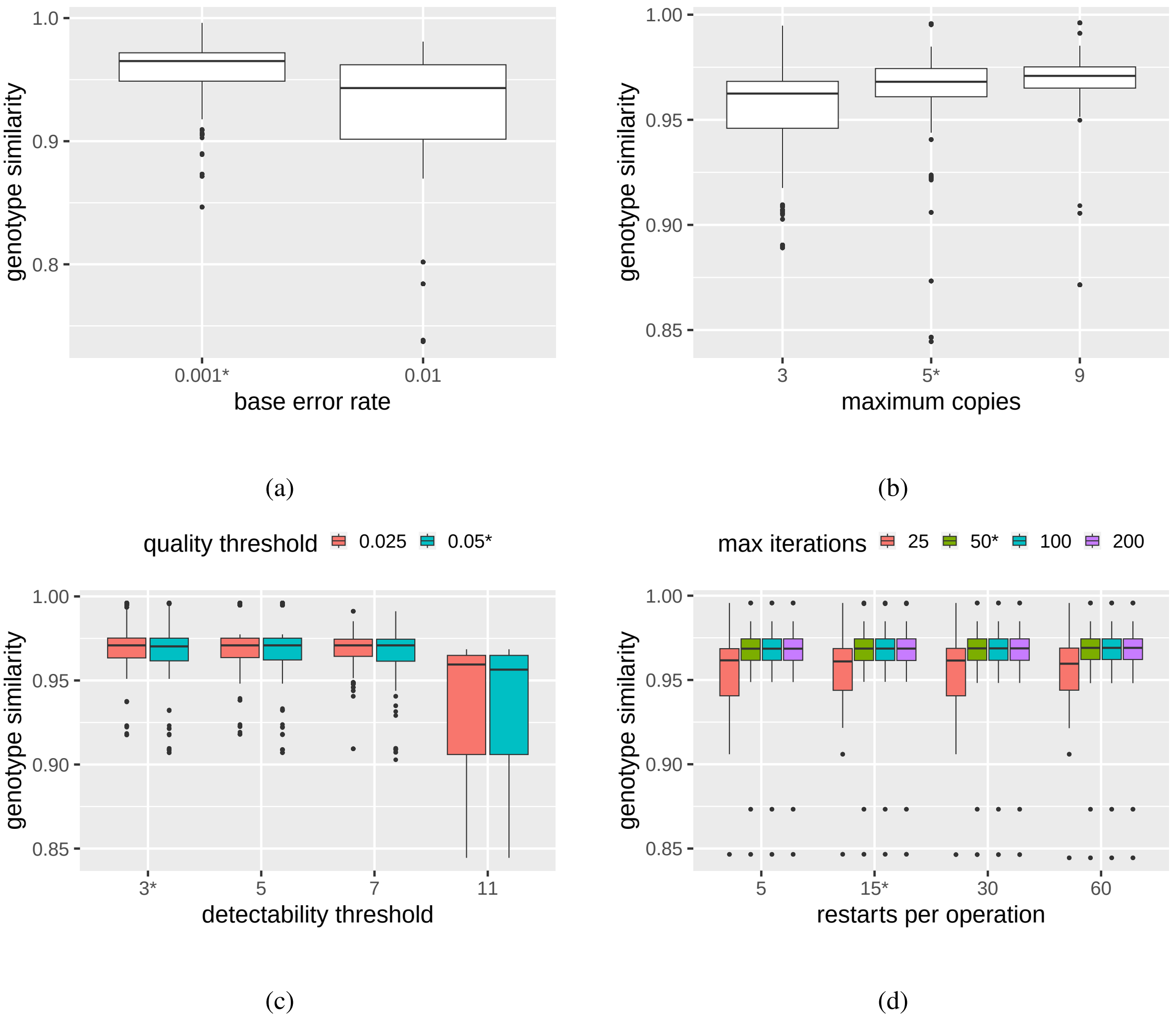

Fig N: **Hyperparameter impact on genotype similarity for $k = 9$ clones, $n = 2000$ cells, $m = 10000$ SNVs and coverage $g = 0.05\times$.** Hyperparameter choices shown with an asterisk (*) indicates the default setting using in simulation study. (a) Impact of hyperparameter base sequence error rate α. (b) Impact of hyperparameter maximum number of copies c with base sequencing error rate held constant at $\alpha = 0.001$. (c) Impact of hyperparameter detection threshold t and quality of extension (qc) threshold (Appendix A.4.6) with base sequencing error rate held constant at $\alpha = 0.001$. (d) Impact of hyperparameter choice on the number of restarts per operation and the maximum number of iterations per operations with base sequencing error rate held constant at $\alpha = 0.001$.

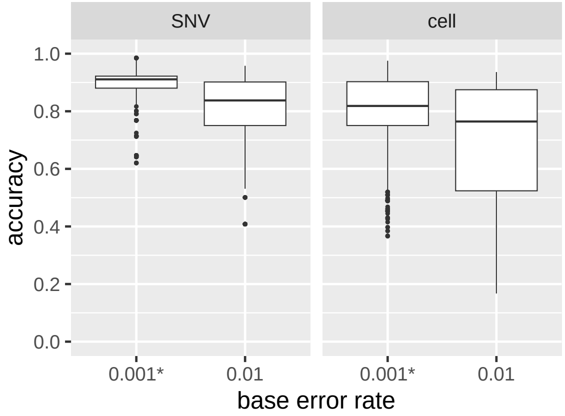

(a)

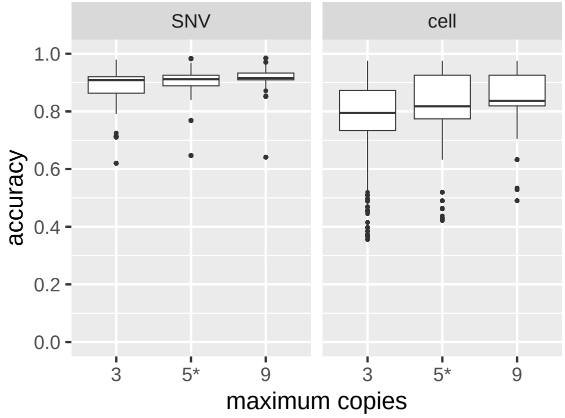

(b)

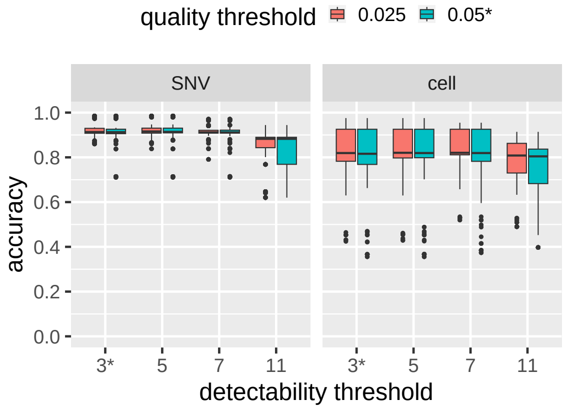

(c)

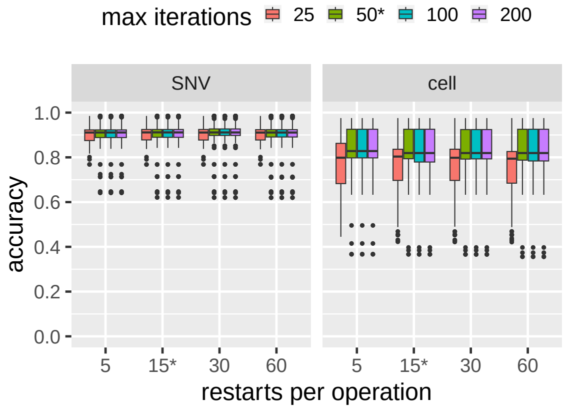

(d)

Fig O: **Hyperparameter impact on tree reconstruction accuracy for $k = 9$ clones, $n = 2000$ cells, $m = 10000$ SNVs and coverage $g = 0.05\times$.** Hyperparameter choices shown with an asterisk (*) indicates the default setting using in simulation study. (a) Impact of hyperparameter base sequence error rate α. (b) Impact of hyperparameter maximum number of copies c with base sequencing error rate held constant at $\alpha = 0.001$. (c) Impact of hyperparameter detection threshold t and quality of extension (qc) threshold (Appendix A.4.6) with base sequencing error rate held constant at $\alpha = 0.001$. (d) Impact of hyperparameter choice on the number of restarts per operation and the maximum number of iterations per operations with base sequencing error rate held constant at $\alpha = 0.001$.

B.2 Experimental data

Below we provide pertinent details and supplemental results for our analysis of high-grade serous ovarian cancer cells sequenced via DLP+ and triple negative breast cancer tumors sequenced via ACT.

- Appendix B.2.1 provides details of the PHERTILIZER runtime parameters used for experimental data.
- Appendix B.2.2 contains amplifying information on how cancer related genes were placed on inferred trees.
- Appendix B.2.3 provides additional description and analysis of the cell mutational burden metric
- Appendix B.2.4 contains supplementary results for the high-grade serous ovarian cancer cells sequenced with DLP+
- Appendix B.2.5 provides the data processing details for the triple negative breast cancer tumors sequenced via ACT
- Appendix B.2.6 contains supplemental results and figures for the triple negative breast cancer tumors sequenced via ACT

B.2.1 PHERTILIZER runtime parameters for experimental data

We set hyperparameters $c = 5$ and $\alpha = 0.001$ for all experimental data analyses. We used 25 restarts and a maximum of 50 iterations for each elementary tree operation. Additionally, we performed a grid search over detectability threshold $t \in \{5, 6, 7\}$ (Appendix A.3) and the lower bound for the quality check qc $\in \{0.05, 0.075\}$ (Appendix A.4.6). The upper bound for the quality check qc was set to 0.15. After running PHERTILIZER on each of these 6 combinations of parameters, we selected the clonal tree with maximum posterior probability.

B.2.2 Placement of driver genes on inferred trees

We annotated inferred clonal trees with cancer-related genes listed in the Cancer Gene Census (CGC) [15] from COSMIC v97 and cBioPortal [16, 17]. In particular, we identified missense variants in cancer-related genes as well as genes with stop-gain variants. We first selected stop-gain variants. Then, we annotated variants with VEP [18], and looked for missense variants that are predicted to be damaging or deleterious by both SIFT [19] and PolyPhen [20]. Among these, we selected variants either present in the CGC, or in more than 0.4% of the total 76,639 samples from 10 pan-cancer studies included in cBioPortal. As a large number of SNVs are found in patient TN8 of the ACT dataset, we only labeled genes present in the CGC.

B.2.3 Cell mutational burden (CMB)

To assess the quality of each inferred clade for real data, we developed a performance metric called *cell mutational burden* (CMB) defined as

$$\mathrm{CMB}(i, M) = \sum_{q \in M} \mathbf{1}\{a_{iq} > 0\} / \sum_{q \in M} \mathbf{1}\{d_{iq} > 0\}. \tag{46}$$

In words, $\mathrm{CMB}(i, M)$ is the fraction of mapped SNV loci M with mapped variant reads in cell i.

For a specified *clade* j or subtree rooted at node v_j, SNVs M_j are the SNVs gained at node v_j. CMB is designed to assess the goodness of fit of a proposed clonal tree without a known ground truth tree and succinctly captures the relationship between the inferred tree, clonal genotyping, and cell clustering. For

a cell i placed within clade j, we expect $\mathrm{CMB}(i, M_j)$ to be high, although the value will depend on copy number. By contrast, for cells placed outside of clade j, we expect $\mathrm{CMB}(i, M_j)$ to be low.

Because this is a newly proposed metric that has complex interactions between inference errors, we performed a sensitivity analysis on a simulated tree (Fig. Pa) with varying rates in $\{0\%, 15\%, 30\%\}$ of cell and SNV placement errors (Fig. P). For reference, we also show cell and SNV placement on the simulated ground truth tree (Fig. Pb) at the highest error rate for both (30%). This analysis showed that in the error free regime, CMB value for cells in clade ranges from from 0.2 to 0.8 but the value will depend on copy number. We also found that as SNV error rates increase, the median CMB for cells outside of the clade increases with median values as high as 0.1 and an increased number of outliers with values as high as 0.7. Finally, as cell error rates increase the variability of CMB values for cells in clade increases. Although this analysis was only performed on a single simulation instance, it is helpful for interpreting this metric on experimental data where ground truth is unknown.

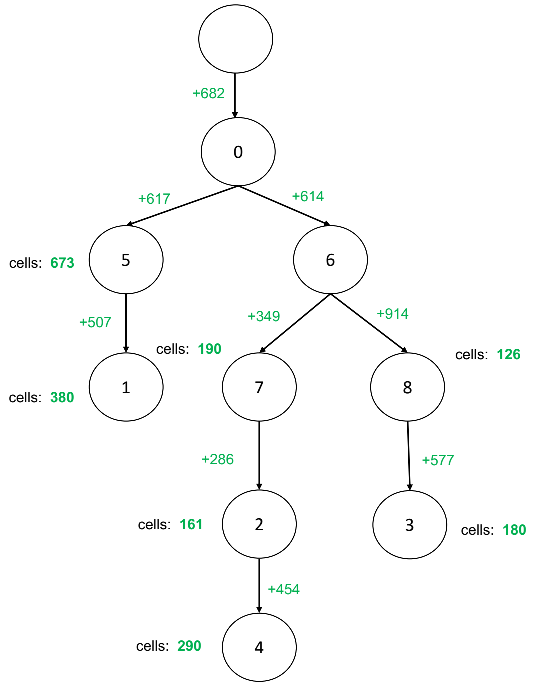

(a) Simulated tree with cell 0% and SNV 0% error rate.

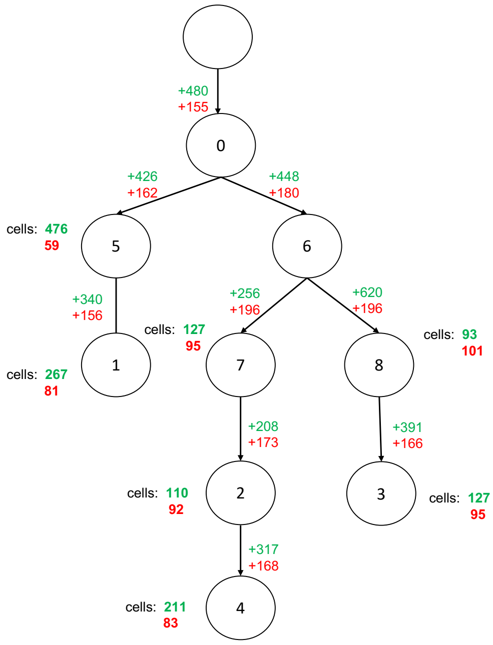

(b) Simulated tree with cell 30% and SNV 30% error rate.

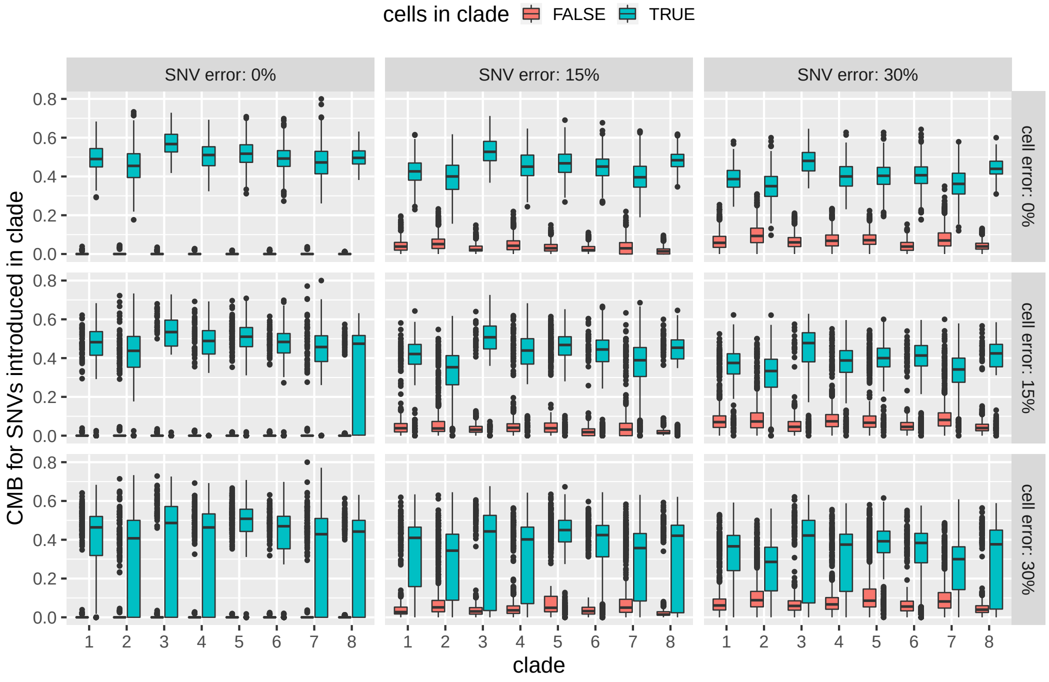

(c) Cell mutational burden (CMB) with varying cell and placement error rates.

Fig P: **Analysis of cell mutational burden (CMB) on a simulated instance with varying error rates in** $\{0\%, 15\%, 30\%\}$ **for both cell and SNV placement.** (a) The simulated ground truth tree with 0% cell placement error rate and 0% SNV placement error rate. (b) The simulated ground truth tree with 30% cell placement error rate and 30% SNV placement error rate. (c) Cell mutational burden (CMB) comparison between cells within (blue) and outside of (red) each clade in the inferred clonal tree at varying cell and SNV placement error rates.

B.2.4 Supplemental figures for high-grade serous ovarian cancer cells sequenced with DLP+

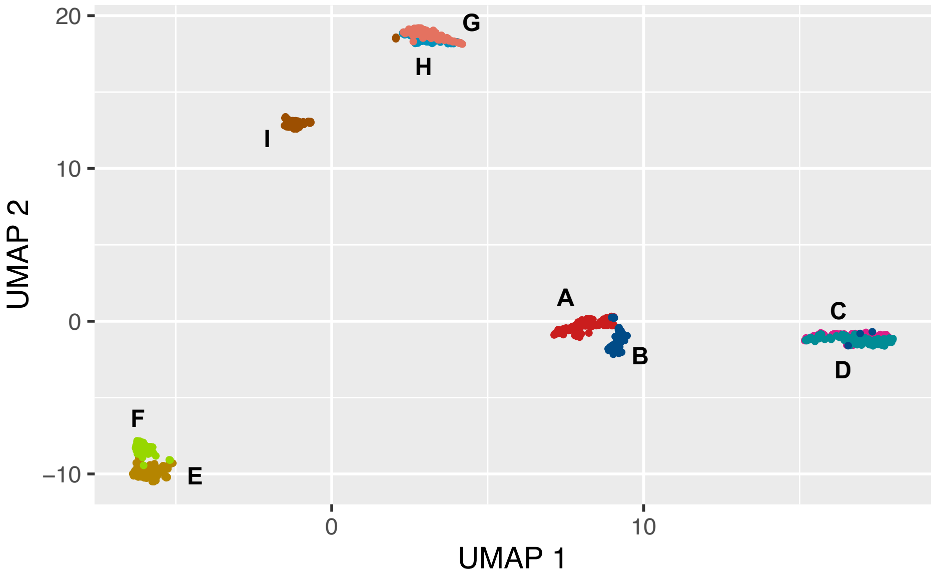

(a) Laks et al. [1] cell clusters

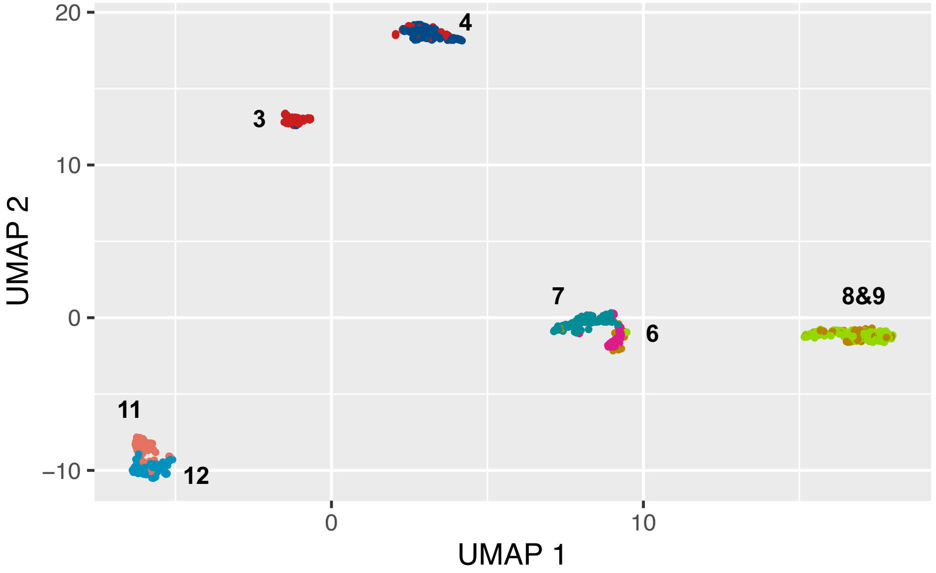

(b) Phertilizer cell clusters

Fig Q: **UMAP for the high-grade serous ovarian cancer patient depicted with inferred cell clusterings.**

B.2.5 Processing the triple negative breast cancer tumors sequencing data from ACT

We merged reads from all cells into a single FASTQ file for each sample, aligned the merged reads to the Human reference genome hg19 (GRCh37) using bowtie2 (v.2.4.4), and sorted by samtools (v.1.15) forming pseudo-bulk samples. We then get a set of SNVs by running Mutect2 [21] in tumor-only mode on each pseudo-bulk sample, followed by FilterMutectCalls in GATK. We further select SNVs whose read depth is greater or equal to 11, VAF greater than 0.033, and have greater or equal to 4 variant reads in the pseudo-bulk sample. In order to match the bins in the original paper, we directly used the normalized read counts from ACT and find the low-dimensional embedding using UMAP [3].

B.2.6 Supplemental tables and figures for triple negative breast cancer tumors sequenced with ACT

- Table A provides a summary of the ACT data of all eight triple negative breast cancer tumors
- Fig. R shows a comparison of PHERTILIZER and Minussi et. al. [2] inferred cell clustering in the embedding space
- Fig. S shows the cell mutational burden (CMB) results for BASELINE+SCITE inferred trees for breast tumors TN3 and TN5
- Fig. T shows PHERTILIZER supplemental results for breast tumor TN2
- Fig. U shows PHERTILIZER supplemental results for breast tumor TN4
- Fig. V shows PHERTILIZER supplemental results for breast tumor TN8

Tumor	Cells	SNVS	Coverage	Phertilizer clones	Minussi et al. super clones	Minussi et al. subclones	SBMclone clones
TN1	1100	13,934	0.031×	6	4	17	1
TN2	1024	8,474	0.039×	6	4	15	1
TN3	1101	13,064	0.020×	1	4	9	1
TN4	1307	14,333	0.022×	5	4	22	1
TN5	1238	10,271	0.020×	1	4	7	1
TN6	1205	11,827	0.017×	1	4	15	1
TN7	915	7,873	0.019×	1	3	18	1
TN8	1224	73,359	0.021×	2	4	15	1

Table A: **Summary of results on the Minussi et al. [2] breast cancer data.** Table depicting the tumor, number of cells and SNVs, the coverage, and the number of clones inferred by different methods for the eight analyzed breast cancer tumors.

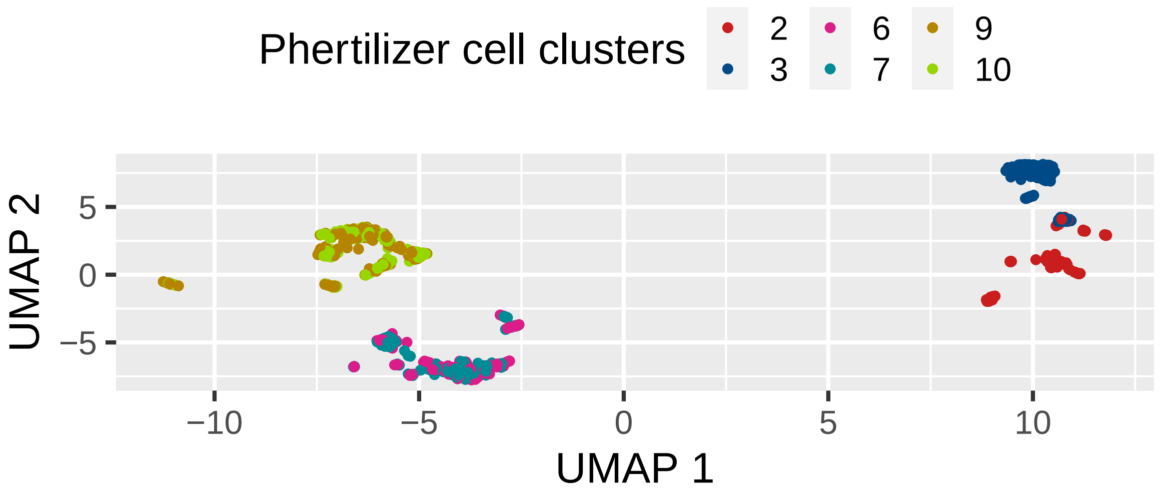

(a) PHERTILIZER UMAP for the triple negative breast tumor TN1.

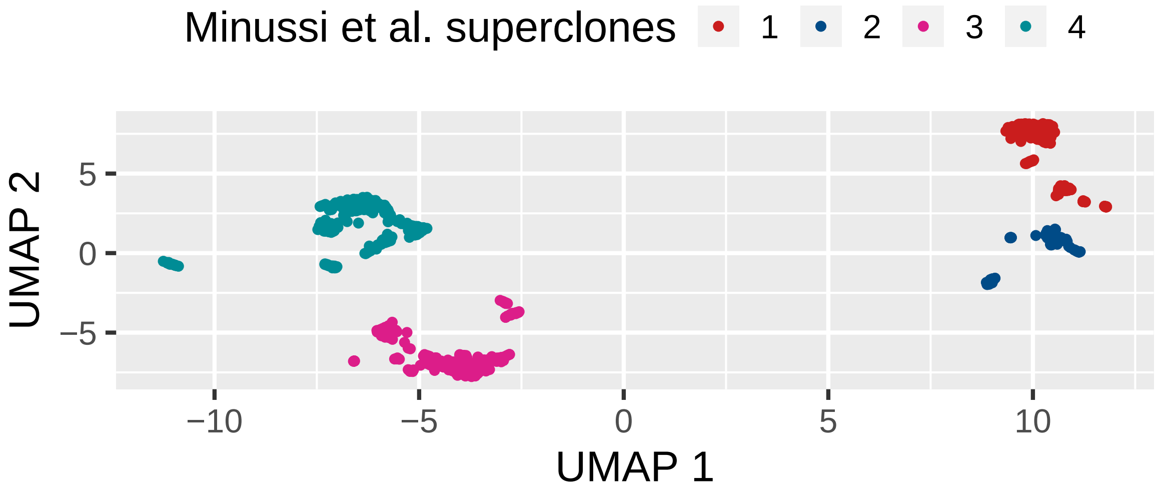

(b) Minussi et al. [2] UMAP for the triple negative breast tumor TN1.

Fig R: **Cell clustering comparison for triple negative breast tumor TN1 in embedding space between PHERTILIZER and Minussi et al. [2]**

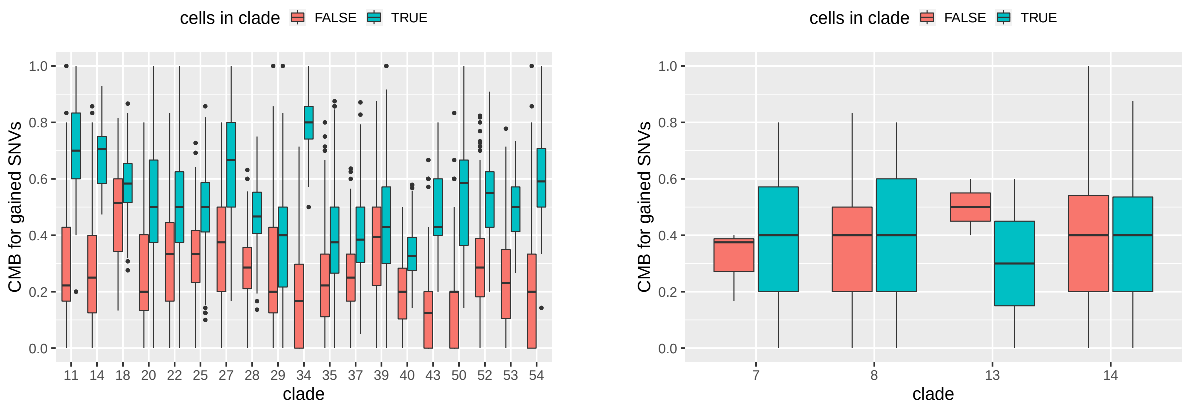

(a) Triple negative breast tumor TN3

(b) Triple negative breast tumor TN5

Fig S: **Cell mutational burden per clade in trees inferred by BASELINE+SCITE for tumors TN3 and TN5**

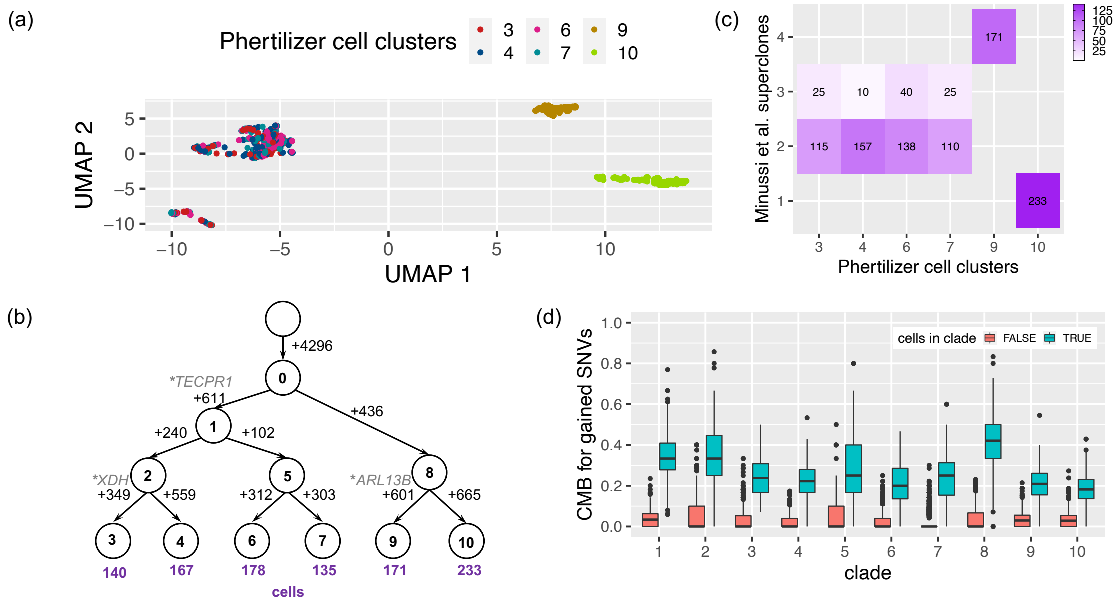

Fig T: **PHERTILIZER inferred clonal tree for breast cancer tumor TN2.** (a) UMAP for the triple negative breast tumor TN2. (b) The tree inferred by PHERTILIZER with numbers of SNVs labeled beside the edges, and numbers of cells labeled beneath the leaves. Cancer-related genes are labeled next to the SNVs ('*': stop-gain variant). (c) A mapping between PHERTILIZER's cell clusters and the Minussi et al. [2] superclones. (d) The CMB distribution for the inferred clades for TN2.

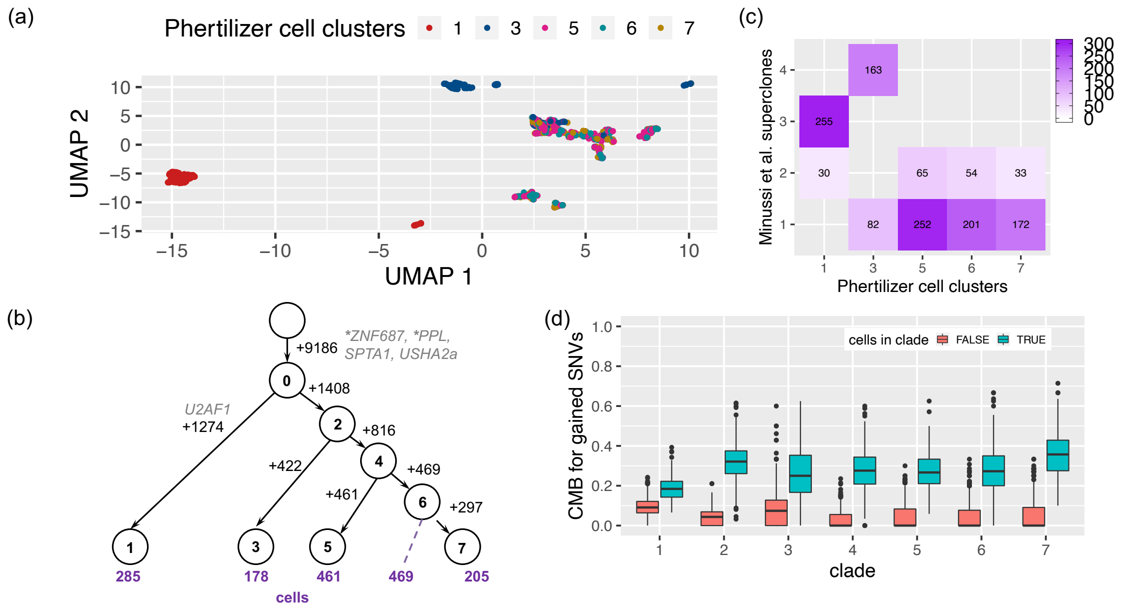

Fig U: **PHERTILIZER inferred clonal tree for breast cancer tumor TN4.** (a) UMAP for triple negative breast tumor TN4. (b) The tree inferred by PHERTILIZER with numbers of SNVs labeled beside the edges, and numbers of cells labeled beneath the leaves. Cancer-related genes are labeled next to the SNVs (‘*’: stop-gain variant). (c) A mapping between PHERTILIZER’s cell clusters and the Minussi et al. [2] superclones. (d) The CMB distribution for the inferred clades for TN4.

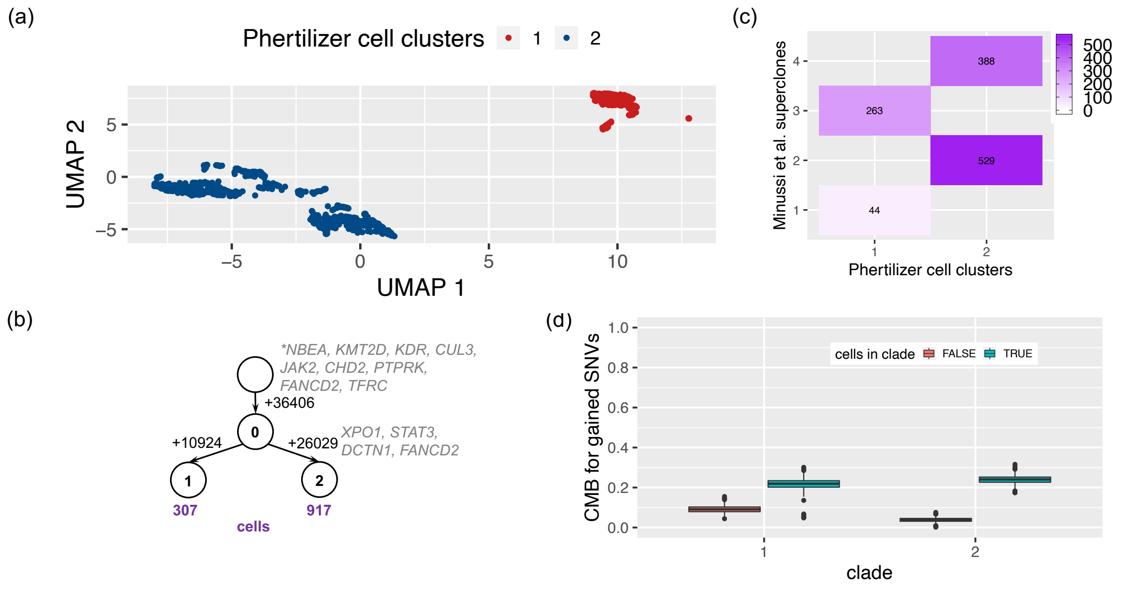

Fig V: **PHERTILIZER inferred clonal tree for breast cancer tumor TN8.** (a) UMAP for triple negative breast tumor TN8. (b) The tree inferred by PHERTILIZER with numbers of SNVs labeled beside the edges, and numbers of cells labeled beneath the leaves. Cancer-related genes are labeled next to the SNVs (‘*’: stop-gain variant). (c) A mapping between PHERTILIZER’s cell clusters and the Minussi et al. [2] superclones. (d) The CMB distribution for the inferred clades for TN8.

References

[1] Laks E, McPherson A, Zahn H, Lai D, Steif A, Brimhall J, et al. Clonal decomposition and DNA replication states defined by scaled single-cell genome sequencing. Cell. 2019;179(5):1207–1221.

[2] Minussi DC, Nicholson MD, Ye H, Davis A, Wang K, Baker T, et al. Breast tumours maintain a reservoir of subclonal diversity during expansion. Nature. 2021;592(7853):302–308.

[3] McInnes L, Healy J, Melville J. UMAP: Uniform manifold approximation and projection for dimension reduction. arXiv preprint arXiv:180203426. 2018;.

[4] Weber LL, Sashittal P, El-Kebir M. doubletD: detecting doublets in single-cell DNA sequencing data. Bioinformatics. 2021;37(Supplement_1):i214–i221.

[5] De Bourcy CF, De Vlaminck I, Kanbar JN, Wang J, Gawad C, Quake SR. A quantitative comparison of single-cell whole genome amplification methods. PloS one. 2014;9(8):e105585.

[6] Aggarwal CC, Hinneburg A, Keim DA. On the Surprising Behavior of Distance Metrics in High Dimensional Space. In: International Conference on Database Theory. Springer; 2001. p. 420–434.

[7] Kriegel HP, Kröger P, Zimek A. Clustering high-dimensional data: A survey on subspace clustering, pattern-based clustering, and correlation clustering. ACM Transactions on Knowledge Discovery from Data. 2009;3(1):1–58.

[8] Shi J, Malik J. Normalized cuts and image segmentation. IEEE Transactions on pattern analysis and machine intelligence. 2000;22(8):888–905.

[9] Pedregosa F, Varoquaux G, Gramfort A, Michel V, Thirion B, Grisel O, et al. Scikit-learn: Machine Learning in Python. Journal of Machine Learning Research. 2011;12:2825–2830.

[10] Damle A, Minden V, Ying L. Simple, direct and efficient multi-way spectral clustering. Information and Inference: A Journal of the IMA. 2019;8(1):181–203.

[11] Ankerst M, Breunig MM, Kriegel HP, Sander J. OPTICS: Ordering points to identify the clustering structure. ACM Sigmod record. 1999;28(2):49–60.

[12] Dollo L. Les lois de l'évolution. Bulletin de la Société belge de géologie, de paléontologie et d'hydrologie. 1893;7:164–166.

[13] Kimura M. The number of heterozygous nucleotide sites maintained in a finite population due to steady flux of mutations. Genetics. 1969;61(4):893.

[14] El-Kebir M, Oesper L, Acheson-Field H, Raphael BJ. Reconstruction of clonal trees and tumor composition from multi-sample sequencing data. Bioinformatics. 2015;31(12):i62–i70.

[15] Sondka Z, Bamford S, Cole CG, Ward SA, Dunham I, Forbes SA. The COSMIC Cancer Gene Census: describing genetic dysfunction across all human cancers. Nature Reviews Cancer. 2018;18(11):696–705.

[16] Cerami E, Gao J, Dogrusoz U, Gross BE, Sumer SO, Aksoy BA, et al. The cBio cancer genomics portal: an open platform for exploring multidimensional cancer genomics data. Cancer Discovery. 2012;2(5):401–404.

[17] Gao J, Aksoy BA, Dogrusoz U, Dresdner G, Gross B, Sumer SO, et al. Integrative analysis of complex cancer genomics and clinical profiles using the cBioPortal. Science Signaling. 2013;6(269):pl1–pl1.

[18] McLaren W, Gil L, Hunt SE, Riat HS, Ritchie GR, Thormann A, et al. The Ensembl variant effect predictor. Genome biology. 2016;17(1):1–14.

[19] Vaser R, Adusumalli S, Leng SN, Sikic M, Ng PC. SIFT missense predictions for genomes. Nature protocols. 2016;11(1):1–9.

[20] Adzhubei I, Jordan DM, Sunyaev SR. Predicting functional effect of human missense mutations using PolyPhen-2. Current protocols in human genetics. 2013;76(1):7–20.

[21] Van der Auwera GA, O'Connor BD. Genomics in the cloud: using Docker, GATK, and WDL in Terra. O'Reilly Media; 2020.
