# Supplementary material for: Phertilizer: Growing a clonal tree from ultra-low coverage single-cell DNA sequencing of tumors
Source: PLoS Comput Biol. 2023 Oct 11;19(10):e1011544. doi: 10.1371/journal.pcbi.1011544 (PMC10593221; doi:10.1371/journal.pcbi.1011544)
Supplement: Attachment — Submitted filename: RECOMB-CCB 2023 Reviews.pdf [file pcbi.1011544.s002.pdf]

RECOMB-CCB 2023 Reviews

SUBMISSION: 562
TITLE: Phertilizer: Growing a Clonal Tree from Ultra-low Coverage Single-cell DNA Sequencing of Tumors

We are extremely appreciative of the helpful feedback that we have received from the reviewers. Our revised manuscript has three main changes in response to reviewer feedback. First, we included an additional analysis on simulated data where we varied model hyperparameters, regularization parameters and runtime parameters to assess performance under different selections and demonstrated that Phertilizer maintains good performance under a range of reasonable values. Second, we attempted to benchmark our method against SCARLET and BiTSC2, which also incorporate copy number information into the tree inference problem. However, both methods failed to run on our simulated data. SCARLET failed to appropriately handle cell and SNV positions with zero read counts, as is common in our data and BiTSC2 was unable to scale to the size of the simulated high-throughput datasets. Lastly, we included additional analyses on the running time of our elementary tree operations used to grow our clonal trees, showing it scales slightly better with increasing numbers of SNV than of cells. This will be beneficial as we noted that future datasets will likely contain a number of SNVs that is orders of magnitudes greater than the number of cells, as evidenced by the datasets analyzed in our work.

---------------------- REVIEW 1 --------------------
SUBMISSION: 562
TITLE: Phertilizer: Growing a Clonal Tree from Ultra-low Coverage Single-cell DNA Sequencing of Tumors
AUTHORS: Leah Weber, Chuanyi Zhang, Idoia Ochoa and Mohammed El-Kebir

----------- Overall evaluation -----------
SCORE: 2 (accept)
----- TEXT:
In this paper the authors present PHERTILIZER, a novel method to infer a clonal tree from ultra-low coverage scDNAseq data. The method, that leverages strong CNA signals to guide the inference of SNV phylogenies, is useful as it aims at interpreting data from state-of-the-art sequencing technologies.

Additionally, I find that the authors have made an appreciable effort to answer the questions and follow the suggestions from previous reviewers. In particular, I think that the formalisation of the method is now presented in a clear way. Since clarity of the method explanation has been of major concern in the previous review, following the introduced improvements I recommend acceptance of this submission.

Thank you for the positive feedback for providing additional helpful suggestions. We are glad to hear that our revisions have been successful in improving the paper.

However, I would still recommend additional revision based on the following comments:

1. Answering point 2. from previous Reviewer 1, the authors claim to have written in Appendix A.2.1 that the model now assumes a flat prior on possible copy numbers. While this is true, they still keep a definition of a non-uniform prior (Equation 3) in the appendix and refer to it in the methods section of supplementary material. Additionally it is not clear if this setting was changed after review and how this change impacts the results.

Thank you for pointing this out. This was indeed an oversight in the description of this probability model. We now write:

> "*Without assuming any prior information on the chromosomal copy numbers in each clone j, this model assumes that the latent VAF x_jq is directly proportional to the number of occurrences in multiset S.*" (Appendix A.2.1)

We emphasize that no changes were made to this setting after the review and so it didn't not impact any of the results.

2. Regarding the clone tree inference algorithm, I think that the elementary operations are now well described and clear. Still, it is not clear to me how these elementary operations are combined (e.g. at each step, which operation among Linear, Branching and Identity is chosen). Additionally, in section A.4.1 the authors state that the recursion of Linear terminates when only the Identity operation can be performed, and that the details of this criterium are presented in section A.4.3. However it is not clear to me where and how this explanation is provided.

We apologize for our oversight regarding the clarity of how the elementary tree operations are combined. To correct this, we have added a new supplemental section (A.4.5) and Supplemental Figure E that describes the growing phase. Briefly, we maintain a candidate set of clonal trees, where each tree is either marked visited or unvisited. Whenever a candidate tree is added to the set it is initially marked as unvisited. After a candidate tree is explored, it is marked as visited. The growing phase is complete when every candidate tree is marked visited. To visit an unvisited tree in the candidate set, we consider the subset of leaf nodes such that each of these leaf nodes is not marked as terminal. A leaf node is marked as terminal if an identity operation was previously applied to that leaf node. We first apply each elementary tree operation to each non-terminal leaf node and store these tree extensions. We then enumerate new unvisited candidate trees by applying all valid sequences of elementary tree operations to the set of non-terminal leaf nodes (see Supplemental Figure E). These new candidate trees are marked as unvisited and added to the candidate set. This phase terminates once all candidate trees in the set have been visited. (A.4.1)

Additionally, we have added a brief supplemental section (A.4.4) that describes our identity operation. An identity operation can be performed on any tree and leaf node and simply returns an unmodified tree, marking the specified leaf node as terminal. It is useful during the growing phase. We have rewritten the confusing sentence to now read:

"The recursion terminates when either of the newly generated clones fails a regularization check, i.e., a detectability check or quality assessment of an extension. See Section A.4.6 for details on these criteria." (Appendix A.4.2)

The description of how these two checks are performed are described in A.4.6 and we hope that these criteria are now easy to locate and follow.

3. In section A.2.2, the authors provide a formula for the Binomial variant read count model. However, they do not provide any explanation for the used success probability formulation.

We have now added the following text to section A.2.2 that describes the success probability formulation for our Binomial variant read count model.

> "Without sequencing error, the success probability for a variant read would be latent VAF x_{jq}. Let \alpha be the probability of misreading a single nucleotide during sequencing. For a successful trial, meaning we observe a variant allele, we have two independent cases. In case I, we have no sequencing error (1-\alpha probability) and x_jq probability of reading a variant allele. In case I we have 1-x_{jq} probability of sequencing a reference allele and \alpha/3 probability of misreading the reference allele for the variant allele." (Appendix A.2.2)

Since my comments address the form of presentation of the method rather than its validity, I evaluate the submission as "accept".

Thank you for the positive assessment.

----------------------- REVIEW 2 ---------------------
SUBMISSION: 562
TITLE: Phertilizer: Growing a Clonal Tree from Ultra-low Coverage Single-cell DNA Sequencing of Tumors
AUTHORS: Leah Weber, Chuanyi Zhang, Idoia Ochoa and Mohammed El-Kebir

----------- Overall evaluation -----------
SCORE: 2 (accept)
----- TEXT:
The paper introduces a novel method called PHERTILIZER for constructing clonal trees of tumor progression using ultra-low coverage single-cell DNA sequencing data. The method was applied to both simulated and real datasets. The algorithmic approach employed by PHERTILIZER seeks to find a tree supported by copy number aberrations and single nucleotide variants (SNVs). The results are promising and demonstrate the practical significance of PHERTILIZER. The paper is well-written and clear to follow. I have only one concern to raise.

Thank you so much for your praise regarding the practical significance of our work as well as the clarity of the writing. We very much appreciate your feedback as well as your additional suggestions to further improve our manuscript.

The authors stated that a comparison with methods that explicitly consider both CNAs and SNVs to retrieve the phylogeny is not possible because they are not conceived for single-cell extreme low-coverage data. However, more is needed to justify comparing the result to only SCITE, which is not designed for low-coverage data nor to exploit CNA. The authors should try to run some of them (e.g., SCARLET or BiTSC2 https://urldefense.com/v3/__https://doi.org/10.1093/bib/bbac092__;!!DZ3fjg!8WHmIrAqWZ_wtmQk5-AUO6R0J2HjJF0vye8ZzP71xmTURmZlWF_9IlkkrxMW_Dhn37oN2QAa_8jNgWlfInu0OtBhYA$), compare the results, or report the computational problems preventing the analyses.

Thank you for the suggestion. We have now attempted to included SCARLET and BiTSC2 in our benchmarking, however both failed to run on our simulated datasets. We now write in line 206 on page 7:

> "*We attempted to include SCARLET[27] and BiTSC2[24] in our benchmarking as these \scs methods incorporate copy number aberrations in their models. However both failed to run on our simulated data. Specifically, SCARLET, which was designed for medium-to-high coverage data, was unable to appropriately handle missing entries in the total read count matrix D. BiTSC2, an MCMC method, was unable to scale to the size of our input datasets --- the largest instances considered in the BiTSC2 paper [24] had $n=500$ cells and $m=200$ SNVs, significantly smaller than our smallest simulation instances with $n=1000$ cells and $m=5000$ SNVs.*" (page 7, line 206)

Moreover, I would suggest further analyses to increase the overall quality of the paper and guide a user to set up PHERTILIZER properly.

1. What is the computational complexity of the method? I noticed that the simulations were run, changing the number of cells and the number of SNVs. I would like to see a formal definition or a discussion to assess which one impact the most and how the approach scale.

In order to help users better understand how Phertilizer scales with the number of cells and SNVs, we included the worst case running time for one elementary tree operation as O(n^3 + nm), where n is the number of cells and m is the number of SNVs on which the operation is being performed. This is the combination of the cell-clustering step (worst case O(n^3)) and the SNV partition step (O(nm)). The details are described in supplement S1 A.4.4 As a result, the algorithm scales slightly better with increasing SNVs than cells. However, we have found Phertilizer to maintain reasonable running times for the size of datasets currently being generated as these datasets, such as the analyzed ovarian and breast cancer datasets, contain a number of SNVs that is orders of magnitudes greater than the number of cells.

Another important factor in the running time is the size of the candidate set of trees, which grows exponentially (O(3^|L(T)|), with each existing unvisited tree in the candidate set where |L(T)| is the number of leaf nodes of the unvisited tree T. We have added a brief discussion to the end of supplement S1 A.4.5 that discusses why the size of the candidate set remains tractable in practice for ultra-low coverage scDNA-seq data. We now write:

> "*Although the size of the candidate set grows exponentially, in practice we find that the size of the candidate set is manageable due to the sparsity of data and regularization (see Section A.4.6 for details). A node tends to become a terminal leaf node after only a few repeated applications of elementary tree operations as one or both of the resulting clones will be undetectable or result in a poor quality extension. This is attributed to either an insufficient number of cells and SNVs that results in the clone falling below the detectablity threshold or because the sequence of operations is such a poor fit to the data that it fails to pass the quality of extension check. These regularization steps, described in the next section, are key to maintaining a manageable candidate set of clonal trees.*" (Appendix A.4.5)

2. What is the impact on hyperparameters (i.e., C and alpha), thresholds (i.e., clone detection and quality assessment of extension), or other parameters (i.e., max number of iterations and the number of restarts) chosen on the results?

Thank you for the helpful suggestion. We performed an additional analysis where we explored the performance of our method when varying the value of hyperparameters c and alpha as well as the detection threshold t, the upper bound for a quality of an extension check, the number of restarts and iterations per elementary tree operation. We included a discussion of this analysis in the main text results section in a paragraph denoted by hyperparameter selection and we included Figures N and O in supplement S1 B.1.5. Overall, we found that Phertilizer is not overly sensitive to reasonable choices of these hyperparameters, maintaining high performance on simulated data. Interestingly, we did observe that the greatest gain in performance occurred as c increases from our default of 5 to 9. We hope that this additional analysis is helpful in guiding users in their selection of hyper and runtime parameters.

Finally, two minor comments:

1. a color scalebar needs to be included in all the heatmaps that compare the cluster assignment (e.g., Fig.4-C, Fig.5-B, etc.).

We have now added a colorbar to Fig. 4C and Fig. 5B as well as Supplemental Figures T,U,V.

2. In section A.4.2, at the beginning of page. 23, the text from "given the SNV partition …" to "… read count embedding R" is repeated from section A.4.1. Please, remove it and refer to the above section.

Thanks. We have modified these sentences with your suggestion.
