# Supplementary material for: Phertilizer: Growing a clonal tree from ultra-low coverage single-cell DNA sequencing of tumors
Source: PLoS Comput Biol. 2023 Oct 11;19(10):e1011544. doi: 10.1371/journal.pcbi.1011544 (PMC10593221; doi:10.1371/journal.pcbi.1011544)
Supplement: Attachment — Submitted filename: Response to Reviewers -- Phertilizer -- PLOS CB.pdf [file pcbi.1011544.s003.pdf]

Phertilizer: Growing a Clonal Tree from Ultra-low Coverage Single-cell DNA Sequencing of Tumors

We are extremely appreciative of the helpful feedback that we have received from the reviewers. Our revised manuscript addresses the critique made by Reviewer #1 regarding acknowledging the rapidly advancing methods for variant calling from single-cell RNA/ATAC-seq technologies. Our point-by-point response is indicated in red color.

Reviewer #1: The authors addressed all concerns in a proper way and, as a result, the paper results more solid.
Overall, the article is methodologically sound and provides an interesting contribution to the field of cancer evolution inference from single-cell data.

My only minor concern is the lack of a proper discussion on the strategies for calling SNVs from single-cell RNA-seq or ATAC-seq data, the availability of which is rapidly growing, particularly thanks to the increasing number of experiments on patient-derived models. See, for instance (but not only):
- Muyas, F., Sauer, C.M., Valle-Inclán, J.E. et al. De novo detection of somatic mutations in high-throughput single-cell profiling data sets. Nat Biotechnol (2023). https://doi.org/10.1038/s41587-023-01863-z
- Ramazzotti, D., Angaroni, F., Maspero, D. et al. Variant calling from scRNA-seq data allows the assessment of cellular identity in patient-derived cell lines. Nat Commun 13, 2718 (2022). https://doi.org/10.1038/s41467-022-30230-w
- Arya R Massarat and others, Discovering single nucleotide variants and indels from bulk and single-cell ATAC-seq, Nucleic Acids Research, Volume 49, Issue 14, 20 August 2021, Pages 7986–7994, https://doi.org/10.1093/nar/gkab621
- Patricia M Schnepp and others, SNV identification from single-cell RNA sequencing data, Human Molecular Genetics, Volume 28, Issue 21, 1 November 2019, Pages 3569–3583, https://doi.org/10.1093/hmg/ddz207
- Liu, F., Zhang, Y., Zhang, L. et al. Systematic comparative analysis of single-nucleotide variant detection methods from single-cell RNA sequencing data. Genome Biol 20, 242 (2019). https://doi.org/10.1186/s13059-019-1863-4

The exploitation of single-cell mutational profiles generated from such data types might allow for the reconstruction of reliable models of cancer evolution, and may represent both an alternative and a complement (in case of multi-modal assays) to scDNA-seq data.

We thank the reviewer for bringing this up. We have included a new paragraph in the Discussion section that recognizes the challenges of variant calling from these technologies while highlighting several recent and promising methods, such as Monopogen, SComatic, VarCA, and scAllele. Following this acknowledgment, we now state that "By accounting for different error profiles in variant calling, Phertilizer could be extended to directly model the evolution of somatic variants from scRNA-seq and ATAC-seq datasets."

Reviewer #2: All the issues pointed out in the RECOMB version have been addressed. I have no other requests.

We thank the reviewer for the positive assessment.
